# Formation of non-spherical polymersomes driven by hydrophobic directional aromatic perylene interactions

**DOI:** 10.1038/s41467-017-01372-z

**Published:** 2017-11-01

**Authors:** Chin Ken Wong, Alexander F. Mason, Martina H. Stenzel, Pall Thordarson

**Affiliations:** 1 School of Chemistry, University of New South Wales, Sydney, NSW 2052 Australia; 2 The Australian Centre for Nanomedicine and the ARC Centre of Excellence in Convergent Bio-Nano Science and Technology, University of New South Wales, Sydney, NSW 2052 Australia; 3 Centre for Advanced Macromolecular Design (CAMD), University of New South Wales, Sydney, NSW 2052 Australia

## Abstract

Polymersomes, made up of amphiphilic block copolymers, are emerging as a powerful tool in drug delivery and synthetic biology due to their high stability, chemical versatility, and surface modifiability. The full potential of polymersomes, however, has been hindered by a lack of versatile methods for shape control. Here we show that a range of non-spherical polymersome morphologies with anisotropic membranes can be obtained by exploiting hydrophobic directional aromatic interactions between perylene polymer units within the membrane structure. By controlling the extent of solvation/desolvation of the aromatic side chains through changes in solvent quality, we demonstrate facile access to polymersomes that are either ellipsoidal or tubular-shaped. Our results indicate that perylene aromatic interactions have a great potential in the design of non-spherical polymersomes and other structurally complex self-assembled polymer structures.

## Abstract (web summary)

Polymersomes have become a powerful tool in drug delivery and synthetic biology, but their use can be restricted by a lack of versatile methods for shape control. Here the authors demonstrate access to a range of non-spherical polymersome morphologies by exploiting hydrophobic directional aromatic perylene interactions within the membrane structure.

## Introduction

Most cells and organelles found in nature adopt highly complex non-spherical shapes that aid the regulation of cell function^1,2^. To study and mimic the mechanisms that govern cellular shape control, polymersomes made of amphiphilic block copolymers, are a promising candidate due to their superior physical and chemical stability^3^. The membrane structure of polymersomes also offers good versatility in the sense that it can be programmed to respond to certain chemical or physical stimuli^4^, or modified with biological components^5,6^. Polymersome formation is largely driven by the hydrophobic effect^7^ which lacks directionality, and in most cases lead to isotropic membrane structures that give rise to their spherical shapes^8–11^. To address this issue, several strategies that have been shown to effectively induce shape transformation of polymersomes to more complex forms (such as tubes, stomatocytes or ellipsoids) include the use of cross-linkers^12^, control over changes in osmotic pressure^13–15^ and the membrane-incorporation of liquid crystalline moieties like cholesterol or phospholipids^16,17^. In most cases, polymersome self-assembly methods only yield one type of shape for a given polymer, e.g., when using rod-coil block copolymers^18^ or the polymerization-induced self-assembly methods^19^, or they require precise control and timing of external forces followed by subsequent trapping of the non-spherical polymersomes, e.g., when using osmotic pressure^13–15^. Ideally, methods for shape-control should be based on self-assembly and capable of generating a range of morphologies, simply by adjusting parameters such as solvent composition. To this end, the interactions driving polymersomes formation should have some directional character.

Aromatic interactions (often controversially^20^ called *π*–*π* interactions^21^) play an important role in both chemical and biological self-assembly^22^. Aromatic interactions are heavily utilized in the design of supramolecular complexes, particularly in the design of artificial donor-acceptor and light-harvesting complexes where large aromatic building blocks including perylenes are used to create elaborate functional structures^23^. When it comes to polymersome design, aromatic interactions have been underutilized^24–26^ despite the fact that they are both directional and highly solvent-dependent^20^, making them exceptionally useful for controlling the self-assembly of amphiphilic block copolymers.

In this work, we demonstrate a supramolecular strategy to fabricate non-spherical polymersomes with anisotropic membranes from polymers bearing perylene^23,27,28^ aromatic side chains. Perylenes were chosen to provide the aromatic interactions in our system due to their strong tendency to aggregate in water, and how their aggregation behavior can easily be probed by means of UV–Vis and fluorescence spectroscopy^29^. The key to this approach is to utilize the directional nature of aromatic supramolecular interactions in combination with their increased strength due to hydrophobicity as the self-assembled structure moves from an organic solvent (tetrahydrofuran (THF)) to water. Our results show that through concentration and solvent changes, we can control the extent of solvation/desolvation of the aromatic perylene surfaces on the polymer, and ultimately introduce anisotropic membrane tension in the polymersome membrane structure, generating the observed ellipsoidal or tubular-shaped polymersomes. Extensive characterization of the polymersomes by means of spectroscopy and microscopy further revealed that not only do these polymersomes possess non-spherical shapes, but also unusual membrane properties. The use of perylene aromatic interactions to direct the self-assembly of amphiphilic polymers is, in our opinion, a straightforward but elegant method for the fabrication of a variety of non-spherical polymersomes and other structurally unusual self-assembled polymer systems.

## Results

### Synthesis

**Fig. 1:**
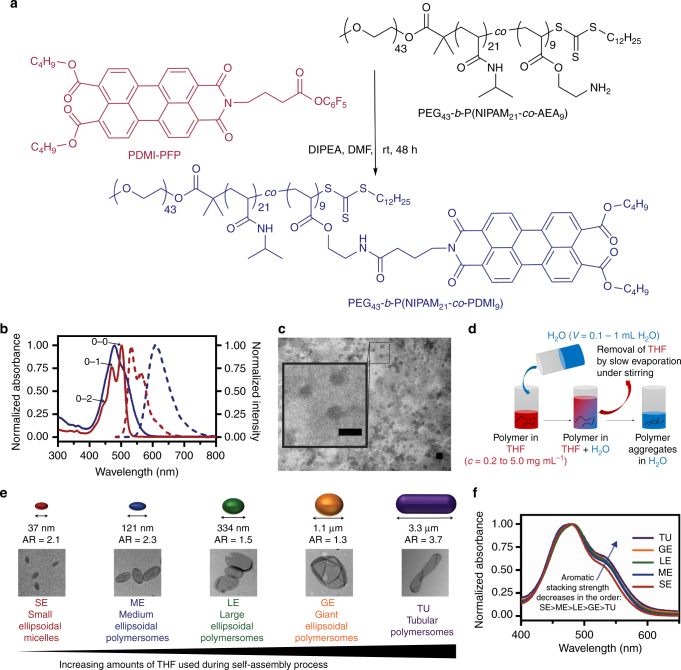
Polymer synthesis, spectroscopic properties and self-assembly. **a** Synthesis of the perylene-bearing diblock terpolymer PEG_43_-*b*-P(NIPAM_21_-*co*-PDMI_9_). **b** Normalized absorption (solid lines) and fluorescence spectra (dashed lines) of the amine-reactive perylene derivative PDMI-PFP (red) and PEG_43_-*b*-P(NIPAM_21_-*co*-PDMI_9_) (blue) in THF. **c** TEM image of spherical aggregates formed by PEG_43_-*b*-P(NIPAM_21_-*co*-PDMI_9_) in a dilute THF solution (0.1 mg mL^−1^). Scale bar, 100 nm. **d** Schematic representation depicting the solvent-switch method used to induce self-assembly of PEG_43_-*b*-P(NIPAM_21_-*co*-PDMI_9_). **e** Schematic representations, average major axis lengths, aspect ratios, and TEM images of resulting self-assembled structures formed at different THF content. **f** Normalized absorption spectra of all five polymersomes in water

To obtain the perylene-bearing diblock terpolymer, poly(ethylene glycol)-*b*-poly(*N*-isopropylacrylamide-*co*-perylene diester monoimide) PEG_43_-*b*-P(NIPAM_21_-*co*-PDMI_9_), we employed a post-polymerization modification strategy involving the attachment of amine-reactive perylene diester monoimide derivatives (PDMI-PFP) onto a diblock terpolymer PEG_43_-*b*-P(NIPAM_21_-*co*-AEA_9_) containing free amines, as shown in Fig. 1a. The polymer PEG_43_-*b*-P(NIPAM_21_-*co*-AEA_9_) was obtained after deprotection of the corresponding *t*-Boc protected PEG_43_-*b*-P(NIPAM_21_-*co*-*t-*BocAEA_9_), synthesized by reversible addition-fragmentation chain transfer (RAFT) copolymerization of *N*-isopropylacrylamide (NIPAM) and *t-*Boc-aminoethyl acrylate (*t*-BocAEA) in the presence of a PEG_43_-modified trithiocarbonate macroRAFT agent (refer to Supplementary Methods for details). The degree of perylene functionalization on the diblock terpolymer was estimated to be 100% based on the ratio of integration of ^1^H-^15^N cross-peaks corresponding to the amide protons of NIPAM and the amide linker protons of PDMI (Supplementary Fig. 1c and Supplementary Table 1). This high degree of functionalization is proposed to be due to the intended spacing out of free amines within the P(NIPAM_21_-*co*-AEA_9_) statistical copolymer block (which ultimately led to a reduction in steric hindrance between side chains). Analysis of the diblock terpolymer by gel permeation chromatography (GPC) (Supplementary Fig. 2) revealed a number-average molecular weight (*M*_n,GPC_) of 15,000 g mol^−1^ and a low dispersity (*Đ*) of 1.09.

### Aggregation in THF

In order to understand the aggregation state of the perylene moieties prior to and after attachment onto the diblock terpolymer, we performed UV–Vis spectroscopy studies (blue and red solid lines, Fig. 1b) on both the amine-reactive perylene diester monoimide derivative PDMI-PFP and perylene-bearing diblock terpolymer PEG_43_-*b*-P(NIPAM_21_-*co*-PDMI_9_) in a good solvent – THF. In the PDMI-PFP absorption spectrum (red solid line), we observed three distinguishable vibronic peaks (corresponding to 0–0, 0–1, and 0–2 transitions) at 501 nm, 471 nm, and 443 nm that can be ascribed to the vibrational modes coupled to electronic S_0_–S_1_ transitions of the perylene chromophore^28,30^. The normal Franck-Condon progression in the PDMI-PFP spectrum with *A*_0–0_>*A*_0–1_>*A*_0–2_ indicates its existence in a non-aggregated (or monomeric) state^28^. Unlike its monomeric counterpart, PEG_43_-*b*-P(NIPAM_21_-*co*-PDMI_9_) displayed a broadened absorption spectrum with inverted intensities for the 0–0 and 0–1 transitions (blue solid line). This inversion in intensities coupled with changes in the *A*_0–0_/*A*_0–1_ value which was calculated to be 0.73, are indicative of the formation of face-to-face aromatic (*π*-stacked) H-aggregates between perylene moieties on the diblock terpolymer (non-aggregated systems typically have an *A*_0–0_/*A*_0–1_≥1.6)^30,31^. The absence of any spectral transition even upon dilution of the diblock terpolymer down to a concentration of 0.017 mg mL^−1^ (Supplementary Fig. 3) suggests very strong intramolecular (intrachain) H-aggregation but no interchain interactions between the perylene units on the polymer.

Next, the fluorescence properties of both PDMI-PFP and PEG_43_-*b*-P(NIPAM_21_-*co*-PDMI_9_) in THF were examined by fluorescence spectroscopy (red and blue dashed lines, Fig. 1a). The monomeric amine-reactive perylene derivative PDMI-PFP displayed a nearly mirror image relationship between the fluorescence and absorption spectra, which is typical of non-aggregated systems^31,32^. However, in the fluorescence spectrum of the perylene-bearing diblock terpolymer PEG_43_-*b*-P(NIPAM_21_-*co*-PDMI_9_), a broad, featureless red-shifted fluorescence that peaked at 607 nm was observed. This apparent loss in vibronic features coupled with the presence of a relatively large Stokes shift (~128 nm) is characteristic of the formation of a low-energy excimer state due to strong *π*–*π* electronic overlap between H-aggregated perylene moieties on the diblock terpolymer^33^.

To investigate the aggregated nature of PEG_43_-*b*-P(NIPAM_21_-*co*-PDMI_9_) in THF, we followed up by combination of synchrotron small-angle x-ray scattering (SAXS) measurements and visualizing the morphology of the aggregates formed using transmission electron microscopy (TEM). The TEM image of the diblock terpolymer drop-casted from a dilute 0.1 mg mL^−1^ THF solution (Fig. 1c) shows the presence of spherical micellar-like particles with diameters in the range of 40–50 nm. At higher concentrations, overcrowding rendered TEM unpractical. The SAXS patterns (Supplementary Fig. 4) for polymer solutions of 0.1 and 0.2 mg mL^−1^ concentrations fitted well to a spherical micelle model. Interestingly, the SAXS patterns for polymer solutions between 0.5 to 5.0 mg mL^−1^ concentrations could not be fitted to the same spherical micelle model, but were instead fitted to a sum model derived using a linear combination of spherical micelle model and lamellar model. These combined results suggest the diblock terpolymer exists solely as spherical micelles only at low polymer concentrations (<0.2  mg mL^−1^). At higher polymer concentrations (>0.5 mg mL^−1^), a mixture of spherical micelles and lamellar structures exists in solution.

### Aggregation in water

While the thermoresponsive polymer PNIPAM^6^ is well known to exhibit a lower-critical solution temperature (LCST) behavior which allows it to reversibly transition between a hydrophilic and hydrophobic state, we found this not to be the case with PEG_43_-*b*-P(NIPAM_21_-*co*-PDMI_9_). The fact that our polymer was completely insoluble even after prolonged stirring in an ice bath strong indicated that the amide groups of NIPAM side chains on our polymer have a hampered ability to form hydrogen bonding interactions with water (i.e., they exist in the hydrophobic state regardless of temperature). We postulate that the amide groups are held together via intra- and intermolecular hydrogen bonding interactions that help to avoid entropic penalty associated with unfavorable solvation of neighboring perylene side chains on the polymer. Note, however, that typical chain collapse observed with PNIPAM is not possible due to presence of the H-aggregated perylenes on our polymer. Our claims above are supported by studies that have shown the susceptibility of PNIPAM’s LCST behavior to changes in the presence of hydrophobic or hydrophilic comonomers^34^. Furthermore, complete losses in LCST behavior, such as in our case, have also been reported before^35,36^.

**Fig. 2:**
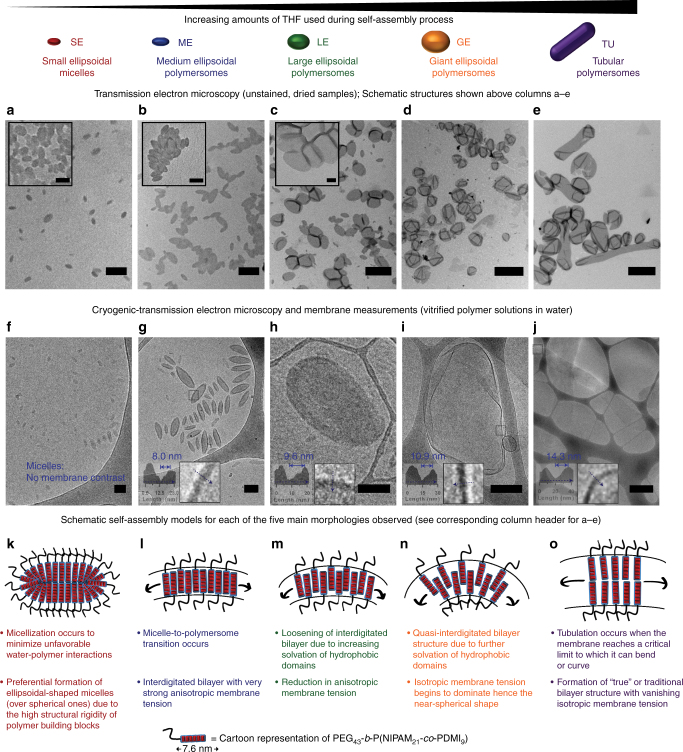
TEM images and proposed self-assembly pathways for the different morphologies observed. **a**–**e** TEM images. The insets in **a**–**c** are higher magnification TEM images. **f**–**j** cryo-TEM images. The insets in **g**–**j** are higher magnification cryo-TEM images and intensity plot profiles used to estimate membrane thickness of individual polymersome morphology. For clarity, an enlarged region of the same cryo-TEM image in **j** is shown in Supplementary Fig. 6. **k**–**o** Schematic representations of the micelle/polymersome structures formed. The black arrows in **l**–**o** indicate the direction of membrane tension. Scale bars in **a**–**j** are 100, 200, 500, 2000, 2000, 50, 100, 100, 300, and 1000 nm, respectively. Scale bars inset in **a**–**c** are 50, 100, and 100 nm, respectively

Since the initially intended temperature-triggered self-assembly strategy was not possible, we instead resorted to the solvent-switch method^37^ to induce self-assembly of the PEG_43_-*b*-P(NIPAM_21_-*co*-PDMI_9_). Briefly, this method, as depicted in Fig. 1d, involves the quick addition of water (poor solvent for the hydrophobic block) into a THF solution of PEG_43_-*b*-P(NIPAM_21_-*co*-PDMI_9_), followed by the removal of THF via evaporation under stirring at ambient conditions (1–3 days). Gas chromatography with flame ionization detection analysis (Supplementary Fig. 5 and Supplementary Table 2) estimates that <0.0025% (v/v) of the original amount of THF added remains in solution after 3 days (if necessary, this residual amount of THF can be removed completely via extensive dialysis against water – see Supplementary Fig. 5e). Using the above-mentioned solvent-switch method, we explored a wide range of THF-water solvent systems and characterized the resulting self-assembled aggregates by TEM (Fig. 2a–e).

As shown in Figs. 1 and 2, we identified five distinct aggregate morphologies that all have anisotropic shapes, namely: small ellipsoidal micelles (SE), medium ellipsoidal polymersomes (ME), large ellipsoidal polymersomes (LE), giant ellipsoidal polymersomes (GE), and tubular polymersomes (TU). These morphologies were attainable through the use of different amounts of THF (volumes ranging from 0.1 to 5 mL) and water (volumes ranging from 0.1 to 1 mL) during the self-assembly process.

**Fig. 3:**
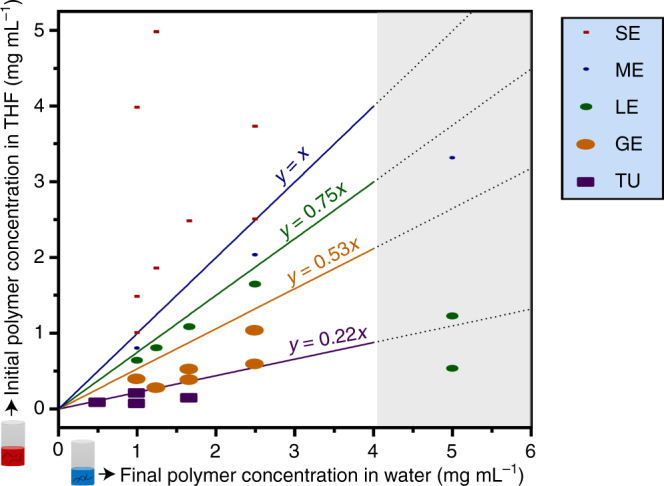
Phase diagram for the self-assembly of PEG_43_-*b*-P(NIPAM_21_-*co*-PDMI_9_) using different THF:water solvent systems. GE, giant ellipsoidal polymersomes; ME, medium ellipsoidal polymersomes; SE, small ellipsoidal micelles; TU, tubular polymersomes. Phase boundaries are represented by straight lines of equation *y* = *mx* + *c* (where *m* = 1, 0.75, 0.53, or 0.22) as depicted in the diagram. The phase boundary lines can be interpreted as follows: in order to obtain ME, one can use any solvent combination that lies within the region between *y* = 0.75*x* (green straight line) and *y* = *x* (blue straight line); e.g., if a final polymer concentration in water of 1 mg mL^−1^ is used, the initial polymer concentration in THF should be between 0.75–1 mg mL^−1^. The gray highlighted region on the right side of the phase diagram indicates a region in which the phase boundary lines become no longer valid due to the tendency of the polymer to precipitate out of solution at low water content

To obtain a better understanding of how variations in solvent quality resulted in these morphologies, we constructed a detailed phase diagram (Fig. 3) based on dominant morphologies that we observed under TEM. The phase diagram is also crucial as it ensures that the morphologies could be reproducibly accessed for further experiments.

The phase diagram elucidates several important aspects that underpin the self-assembly behavior of PEG_43_-*b*-P(NIPAM_21_-*co*-PDMI_9_). First, the morphology of aggregates formed is dictated by both the initial polymer concentration in THF before the addition of water (*y*-axis, Fig. 3) and the final polymer concentration in water after THF evaporation (*x*-axis, Fig. 3). As evidenced from the second left column of the phase diagram, if only a small amount of THF is used to dissolve the polymer and a relatively large amount of water is added to the polymer solution in THF (i.e., above the blue phase boundary line *y* = *x* in Fig. 3, where *x* = 1 and *y* > 1), we obtain small aggregates like SE (Fig. 2a). However, if the initial polymer concentration in THF is decreased to below 1 mg mL^−1^ while the final polymer concentration in water is maintained at 1 mg mL^−1^ (i.e., below the blue phase boundary *y* = *x* in Fig. 3, where *x* = 1 and *y* < 1), then larger aggregates such as ME, LE, GE, and TU (as shown in Fig. 2b–e) are obtained. Second, as we progress from the left to right hand side of the phase diagram (i.e., from low to high final concentration of polymer in water), the morphological transition window for the formation of the large aggregates (ME, LE, GE, and TU) broadens significantly. Third, the phase boundaries become invalid at high polymer concentration in water (i.e., above *x* = 4, as indicated by the gray highlighted region in Fig. 3.) due to the high tendency of the polymer to precipitate out of solution at low water content after THF evaporation (or in other words, this is due to excessive concentrations of the polymer in water). For the same reason, larger structures like GE and TU also appear to become inaccessible within this region.

Next, we performed statistical analyses (*n* = 50) of the populations of these differently shaped aggregates on the basis of our TEM observations using an open source image processing software (ImageJ)^38^. Using the data obtained, we constructed representative histograms (Supplementary Figs. 7–11) for the major axis length, minor axis length, and aspect ratio distributions for the five different morphologies. A summary of the average major/minor axis lengths and average aspect ratios is shown in Table 1. Both the smaller elliptical aggregates (SE and ME) were found to have average aspect ratios of ~ 2.2. Interestingly, however, the larger aggregates (LE and GE) have much lower aspect ratios approaching ~1.4, suggesting they are tending towards a near-spherical shape but should still be considered to be elliptically shaped since spherical particles would have an average aspect ratio of 1.0. The largest aggregates (TU) have the form of tubes with the highest average aspect ratio of ~3.7. The reasons for these differences will be thoroughly discussed later. Note that dynamic light scattering (DLS) analysis which is typically used to determine particle size distribution in solution was also performed (Supplementary Fig. 12), however, the measurements provided unreliably underestimated hydrodynamic diameters due to rotational diffusion (i.e., tumbling motion) of our non-spherical particles in solution. Distinct differences in the overall size of all five morphologies, however, can be seen from the DLS results and may therefore be used to determine the morphologies under bulk conditions if required.

**Table 1:** Measured geometric parameters and *A*_0–0_/*A*_0–1_ values for all five morphologies observed

Morphology	*L*_major_^a^	*L*_minor_^b^	AR^c^	*d*_membrane_^d^	*A*_0–0_/*A*_0–1_^e^
	(nm)	(nm)		(nm)	
SE	37 ± 7	18 ± 5	2.1 ± 0.4	-	0.544
ME	121 ± 32	54 ± 16	2.3 ± 0.2	~8.0	0.590
LE	334 ± 44	230 ± 39	1.5 ± 0.2	~9.6	0.616
GE	1118 ± 237	873 ± 205	1.3 ± 0.2	~10.9	0.621
TU	3396 ± 1125	1039 ± 229	3.7 ± 2.2	~14.3	0.649

^a,b^ *L*_major_ and *L*_minor_ = average (±SD) major and minor axis lengths, respectively

^c^AR = average (±SD) aspect ratios determined by TEM

^d^ *d*_membrane_, membrane thicknesses estimated by cryo-TEM

^e^ *A*_0–0_/*A*_0–1_ = ratio between the *A*_0–0_ (~531 nm) and *A*_0–1_ (~480 nm) UV–Vis absorption values of the self-assembled structure in water

To rule out the possibility that these non-spherical morphologies were shape artefacts introduced during the drying process in conventional drop-casting TEM specimen preparation, we further characterized the aggregates in their frozen-hydrated state using cryogenic TEM (cryo-TEM). As shown in Fig. 2f–j, the aggregate shapes observed under cryo-TEM are in good agreement with those observed under conventional TEM. Statistical analyses based on our cryo-TEM observations were not performed because we noticed that the cryo-TEM analysis was biased towards smaller aggregates since larger aggregates have a higher tendency of being removed during the grid-blotting step.^39^ The vesicular nature of ME was confirmed by the presence of dark outer lines and inner contrast^40^ (corresponding to the membrane and core structure, respectively) surrounding the aggregates shown in Fig. 2g. Evidence of LE, GE, and TU being vesicular, on the other hand, was obtained through the observation of folds and creases around the aggregates^41^ (another common feature of vesicular structures; see Fig. 2h–j).

### Membrane analysis

The membrane thicknesses of ME, LE, GE, and TU were estimated based on their respective intensity plot profiles obtained following a known literature protocol^42,43^ as shown inset in the cryo-TEM images in Fig. 2g–j. Interestingly, all four vesicular morphologies were found to possess different membrane thicknesses (values shown in Table 1), showing that PEG_43_-*b*-P(NIPAM_21_-*co*-PDMI_9_) appears to have different modes of arrangement within different membrane structures, presumably due to the combination of directional and hydrophobic aromatic interactions between the perylene units as will be discussed below. Note, the membrane thicknesses estimated above correspond only to the aggregated hydrophobic P(NIPAM_21_-*co*-PDMI_9_) blocks because the PEG blocks are invisible to electron beam and hence generate poor contrast under electron microscopy^44^.

**Fig. 4:**
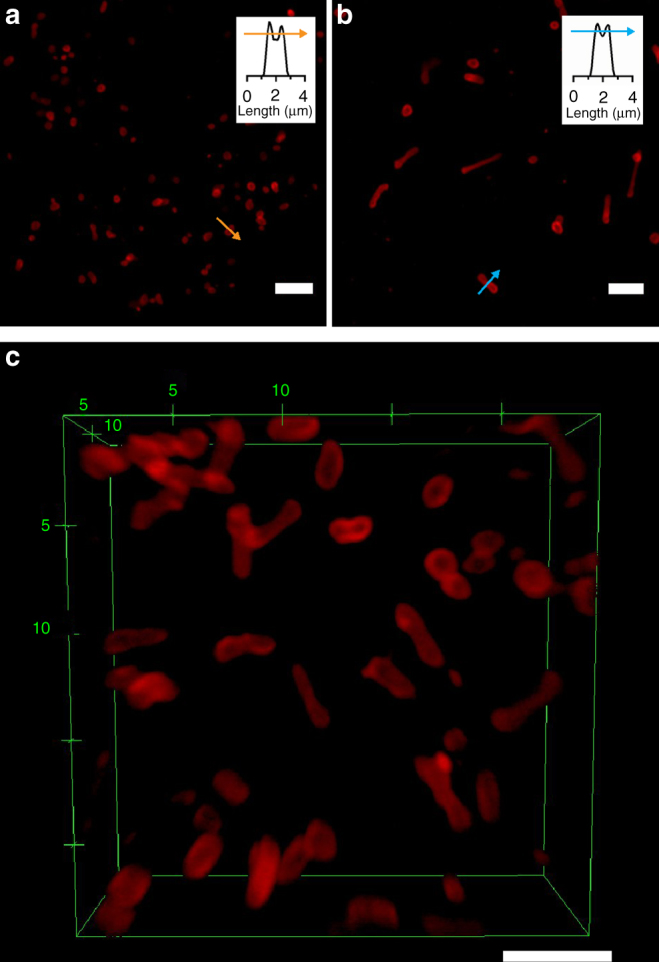
Confocal laser scanning microscopy (CLSM) images of **a** giant ellipsoidal polymersomes (GE) and **b** tubular polymersomes (TU) embedded in 1.5% w/v agarose gel. **c** *Z*-stack image of tubular polymersomes (TU). Insets in **a**, **b** show fluorescence intensity plot profiles highlighting the membrane structure and GE and TU, respectively. Scale bar, 5 µm

The intrinsic fluorescent properties of the aggregates enabled us to perform further structural analysis by confocal laser scanning microscopy (CLSM). Figure 4a, b shows the fluorescence images of GE and TU, respectively. Figure 4c, on the other hand, shows a 3D reconstruction of *z*-stack images of TU. Observations of two distinguishable maxima (corresponding to the membrane structure) in the fluorescence intensity plot profiles shown inset in Fig. 4a, b further confirms the vesicular morphology of both GE and TU. The smaller aggregates (SE, ME, and LE) were not characterized using CLSM as their sizes approach the diffraction limit of light (~250 nm), making it impossible to resolve their membrane structure.

### Aromatic (perylene) interactions in the final structures obtained

As aforementioned, the absorption spectra of perylene chromophores (or more specifically, their *A*_0–0_/*A*_0–1_ values) are highly sensitive to their aggregation state. Therefore, in attempt to understand the supramolecular membrane assembly of PEG_43_-*b*-P(NIPAM_21_-*co*-PDMI_9_), we probed all five morphologies (SE, ME, LE, GE, and TU) in water by UV–Vis spectroscopy (Fig. 1f). The calculated *A*_0–0_/*A*_0–1_ values for each of the morphologies are shown in Table 1. Unsurprisingly, the *A*_0–0_/*A*_0–1_ values for all five morphologies (*A*_0–0_/*A*_0–1_ < 0.65) were found to be lower than that of the pre-assembled diblock terpolymer in THF (*A*_0–0_/*A*_0–1_ = 0.73), indicating that the use of water during the self-assembly process led to more pronounced aggregation of perylenes on the diblock terpolymer.

As can be seen in Fig. 1f, the absorption bands ~531 nm, corresponding to the 0–0 transitions in perylenes, increased in the order of SE, ME, LE, GE to TU. These results reflect that the aromatic (*π*–*π* stacking) interactions between the perylene units on the polymer are strongest in SE and gradually weakens in the larger aggregates. In contrast to the absorption spectra, minimal changes were found in the fluorescence spectra of all the differently shaped aggregates (Supplementary Fig. 13), suggesting that the perylene excimer formation is limited to only within a single diblock terpolymer chain (i.e., no interchain excimer formation occurs). Comparison of the fluorescence spectra of the aggregates to the fluorescence spectrum of the diblock terpolymer in THF (Supplementary Fig. 13) reveals a fluorescence red-shift of 25 nm, which again is indicative of the more pronounced aggregation between perylene units in water than in THF, as discussed above.

### Self-assembly model

Taking into account the evidences from our spectroscopy studies (Fig. 1f) and cryo-TEM analysis (Fig. 2g–j), some relationship clearly exists between the degree of aromatic interactions and membrane thicknesses observed in the different vesicular morphologies. To recap, the smallest vesicular aggregate (ME) possessed the thinnest membrane (~8 nm) in which the diblock terpolymers located in it are held together by the second-strongest (followed by the membrane-less SE micelles) aromatic interactions as shown by the UV–Vis results (Fig. 1f). Conversely, the largest vesicular aggregate (TU) has a membrane with almost double the thickness than that of ME (~14.3 nm), but the diblock terpolymers within the membrane are held together by the weakest aromatic interactions (relative to the other three vesicular morphologies). We propose that, the non-spherical morphologies of our polymersomes are governed by a balance of anisotropic and isotropic membrane tension components^45^, both of which are largely influenced by the packing arrangement of the diblock terpolymers within the membrane structure.

Depending on the initial concentration in THF, the polymer forms either micelles or lamellar structures, the latter dominating at higher concentration according to SAXS measurements (Supplementary Fig. 4). In line with how naphthalene-diimide appended polyurethane self-assembles in methylcyclohexane^26^, we postulate here that the micellar and lamellar structures are formed due to the combination of solvophobic hydrogen bonding interactions between the NIPAM units and aromatic interactions between the perylene units, with the PEG blocks facing the THF solvents, and micellar morphologies dominating at low concentration to minimize edge-interactions with the solvent.

The self-assembly pathway through which PEG_43_-*b*-P(NIPAM_21_-*co*-PDMI_9_) undertakes going from THF to water appears to be dictated by the quality of the solvent system (i.e., THF:water ratio) used in the polymersome fabrication process. In all five cases, the self-assembly processes are initiated similarly, that is, through the quick addition of water (poor solvent) to a THF solution (good solvent) containing the diblock terpolymer. Noting that the polymer is already self-assembled in THF (Fig. 1c), this initial water-addition step induces a cooperative self-assembly process whereby the hydrophobic P(NIPAM_21_-*co*-PDMI_9_) blocks of individual diblock terpolymers spontaneously rearrange and aggregate with one another in order to minimize free energy penalty arising from the increased unfavorable solvophobic (hydrophobic) interactions on moving from THF to water. It should be mentioned that polymersome formation does not occur immediately after the quick water-addition step, but instead forms progressively during the THF-evaporation step as the solvent gradually decreases over time (Supplementary Fig. 14).

In order to simplify our explanations below, we assume that the amount of water used to induce self-assembly is fixed at 500 µL. The addition of this fixed amount of water to a diblock terpolymer solution of low THF content (concentrations >1 mg mL^−1^; for clarity, refer to the region above the phase boundary line *y* = *x*, where *x* = 1 and *y* > 1 in the phase diagram shown in Fig. 3) results in small ellipsoidal micellar aggregates (SE). The formation of micellar assemblies as such is favoured due to presence of large amounts of poor solvent, in this case water, which forces perylene moieties of the diblock terpolymers to pack tightly through very strong hydrophobic and directional aromatic interactions^20^. Based on previous studies, the anisotropic shape of SE is hypothesized to arise from the rigidity of the pre-aggregated perylene units on the polymer in THF (lamellar) where the high edge energy becomes even more unfavorable upon dilution in water^46^. As a consequence, polymer chains around the lamellar edge rearrange to form anisotropic SE structures.

The addition of the same amount of water to a diblock terpolymer solution with slight increment in THF content (concentrations between 1 and 0.75 mg mL^−1^; for clarity, refer to the region in phase diagram (Fig. 3) between phase boundary lines of *y* = *x* and *y* = 0.75*x* where *x* = 1 and 1 > *y* > 0.75) results in medium-sized ellipsoidal polymersomes (ME). Although structurally different from SE (i.e., vesicular vs. micellar), ME too exhibits shape anisotropy in the membrane structure. Comparison of the theoretical fully extended length of the hydrophobic block of the diblock terpolymer (~7.6 nm) with the estimated membrane thickness based on cryo-TEM (~8.0 nm) suggests that the hydrophobic blocks that make up the membrane of ME do not organize themselves in a typical bilayer fashion but instead assembles to form a highly interdigitated membrane structure^42^ with strong anisotropic membrane tension, as depicted in Fig. 2l.

At higher THF content (concentrations between 0.75 and 0.22 mg mL^−1^; for clarity, refer to the region between phase boundary lines of *y* = 0.75*x* and *y* = 0.22*x*, where *x* = 1 and 0.75 > *y* > 0.22 in the phase diagram shown in Fig. 3), we move towards larger structures like large-sized ellipsoidal polymersomes (LE) and giant-sized ellipsoidal polymersomes (GE) that are stabilized by weaker aromatic interactions (Fig. 1f). The increase in membrane thickness of LE and GE (relative to ME; see Table 1) suggests that the polymersome membrane transitions from being in a tightly packed interdigitated state (ME – Fig. 2l) to a much loosely-packed interdigitated state (LE – Fig. 2m), followed by a quasi-interdigitated state (GE – Fig. 2n) as the amount of THF used is increased. These membrane rearrangement phenomena not only increased the membrane thickness but also had apparent effects on the aspect ratio and overall size of the polymersomes (Table 1). The decrease in aspect ratio upon progressing from ME (~2.3) to GE (~1.3) is indicative of changes in the relative contributions of anisotropic and isotropic membrane tension components, with the former being more dominant in LE and the latter contributing more in GE. This is evidenced by the fact that the shape of the polymersomes transitions being anisotropic (ellipsoidal) to isotropic (near-spherical). The significant increase in overall size upon transitioning from ME to GE, on the other hand, is likely a consequence of the increase in isotropic membrane tension component since an increment in size helps to relieve the membrane from unfavorable curvature-induced stress.

At very high THF content (concentrations <0.22  mg mL^−1^; for clarity, refer to the region below the phase boundary line *y* = 0.22*x*, where *x* = 1 and *y* < 0.22 in the phase diagram shown in Fig. 3), we observe a transition from near-spherical GE to tubular-shaped TU (no spherical polymersome formation was observed), indicating that there exists a critical limit to which the membrane may bend or curve. If the membrane curvature were to exceed this said limit, then tubulation via a fusion process would occur. This hypothesis is supported by studies that have shown the promotion of fusion events in both naturally occurring lipid vesicles^47,48^ and polymersomes^25,26^ due to unfavorable membrane tension generated from extreme curvature. On the other hand, the doubled membrane thickness observed in TU (~14.3 nm) relative to in ME (~8 nm) suggests that the polymersome membrane components initially exist in (quasi-)interdigitated states (ME, LE, GE – Fig. 2l–n) before finally rearranging to form a rigid true bilayer (TU – Fig. 2o) in the form of tubes of vanishing isotropic membrane tension. We further note that there may also be a possibility of TU formation being aided by an extrusion effect, caused by hydrodynamic shear forces that are generated by stirring during the solvent-switch process.

### Thermotropic behavior

Upon closer inspection of the cryo-TEM image of ME (Fig. 2g), we found that structures were not exactly ellipsoidal, but instead have a spindle shape, which is characteristic of nematic liquid crystal droplets known as tactoids^45,49^. This observation prompted us to investigate the mesophase (liquid crystalline) properties of our diblock terpolymer by thermal analysis using differential scanning calorimetry (DSC). Indeed, PEG_43_-*b*-P(NIPAM_21_-*co*-PDMI_9_) in the bulk solid state was found to undergo two thermotropic phase transitions upon heating (Supplementary Fig. 15); a first endothermic melting point (*T*_m_) at 52 °C which can be ascribed to a mesophase formation, and a second broad endothermic clearing point (*T*_c_) centered about 116 °C that can be assigned to a mesophase-to-isotropic transition. Note that mesophase formation in our polymer is not entirely unexpected since various perylene derivatives have reportedly been shown to exhibit mesophase properties due to their extensive aromatic stacking character^50–52^.

Following thermal analysis by DSC, we performed further thermal (annealing) studies on the polymersome morphologies in solution (containing small amounts of plasticizing solvent THF) in order to study the thermotropic phase behavior of the diblock terpolymer in its self-assembled polymersome state. Interestingly, we found that the annealing of all our polymersome structures (ME, LE, GE, and TU) at 85 °C for 90 min led to a transformation from anisotropic (non-spherical) polymersomes into purely isotropic (spherical) polymersomes (Supplementary Fig. 16).

The above-mentioned results reveal that the diblock terpolymers within the polymersome membrane most likely exists in the mesophase at room temperature and can undergo a mesophase-to-isotropic transition at much lower temperatures (~85 °C) than as observed by DSC in the bulk solid state (*T*_c_ = 116 °C; refer back to Supplementary Fig. 15) due to the role of THF as a plasticizer which helps to lower both the *T*_m_ and *T*_c_ of the polymer. Furthermore, all four of our non-spherical polymersome morphologies (ME, LE, GE, and TU) represent metastable, kinetically trapped structures that can be converted into more thermodynamically favorable structures (spherical polymersomes; see again Supplementary Fig. 16) via thermal processing above the *T*_m_ of the polymer building blocks in the presence of plasticizing solvent THF.

### Importance of aromaticity and robustness of methodology

To demonstrate the importance of aromaticity and to assess the robustness of our system, we synthesized two additional control polymers; a lesser aromatic negative control polymer, namely PEG_43_-*b*-P(NIPAM_22_-*co*-PDMI_5_) which has approximately half the perylene content of the original polymer (i.e., 5 perylenes per polymer chain as opposed to 9 perylenes per polymer chain) and a more aromatic positive control polymer PEG_43_-*b*-P(NIPAM_23_-*co*-PDMI_19_) which has approximately double the perylene content of the original polymer (i.e., 19 perylenes per polymer chain as opposed to 9 perylenes per polymer chain). Details on the synthesis and characterization of both control polymers are provided in the Supplementary Methods section. Spectral studies in THF (Supplementary Fig. 17) revealed that the perylene moieties on PEG_43_-*b*-P(NIPAM_22_-*co*-PDMI_5_) and PEG_43_-*b*-P(NIPAM_23_-*co*-PDMI_19_) are held together by both weaker and stronger aromatic stacking interactions, respectively, relative to those on PEG_43_-*b*-P(NIPAM_21_-*co*-PDMI_9_).

When subjected to the similar self-assembly process used to fabricate our non-spherical polymersomes (i.e., the solvent-switch method), the lesser negative control aromatic polymer PEG_43_-*b*-P(NIPAM_22_-*co*-PDMI_5_) was found to be unable to generate polymersome structures, but instead self-assembles to form either spherical micelles or worm-like micelles of varying lengths, as shown in Supplementary Fig. 18. As further negative control, we also performed similar self-assembly studies on the non-aromatic precursor polymer PEG_43_-*b*-P(NIPAM_21_-*co*-*t*-BocAEA_9_) and found that this particular polymer does not form polymersome structures too (Supplementary Fig. 19). The two combined negative control results above suggest that aromatic stacking interactions (and, to some extent, an increase in hydrophobicity) plays a crucial role in driving the formation of our anisotropic polymersome structures. The more aromatic positive control polymer PEG_43_-*b*-P(NIPAM_23_-*co*-PDMI_19_), on the other hand, was found to self-assemble into polyhedral-shaped polymersomes (Supplementary Fig. 20; note that these structures are sometimes referred to as faceted vesicles^53,54^ in literature), hence demonstrating the robustness of our methodology in fabricating non-spherical polymersomes. Further studies on how and why these facets form are ongoing and will be the subject of future publication.

In summary, we demonstrated that aromatic interactions can be exploited to direct hydrophobic interactions between aromatic-bearing polymers to generate polymersomes with non-spherical shapes. We show that these polymersomes with anisotropic membrane structures can be tuned into various shapes and sizes simply by controlling the extent of solvation/desolvation of the aromatic-bearing polymer buildings blocks, and how solvent quality affected its spatial arrangement within membranes of different polymersome morphologies. We foresee the use of our methodology to prepare other complex-shaped polymer particles, either by changing the block length of the diblock terpolymer (which in turn affects the rigidity and aromatic association of these building blocks) or by incorporating other substituted perylene or related rylene and pyrene derivatives to induce J-type aggregation instead of H-type aggregation which we have presented here. It is also postulated that our strategy may be extended to prepare higher-order multicompartment structures using ABC triblock terpolymers^55,56^ bearing perylene moieties, as our strategy allows for the introduction of unconventional physicochemical parameters such as aromatic interaction strengths, stacking geometries and rigidity. We also envision that our strategy for forming non-spherical polymer assemblies could have potential applications in drug delivery^57^.

## Methods

### Synthesis of PEG_43_-*b*-P(NIPAM_21_-*co*-PDMI_9_)

The *t*-Boc deprotected diblock terpolymer PEG_43_-*b*-P(NIPAM_21_-*co*-AEA_9_) (86 mg, 0.015 mmol), *N*-(pentafluorophenyl butyl ester)-perylene-3,4,9,10-tetracarboxylic monoimide dibutyl ester (PDMI-PFP, 158 mg, 0.200 mmol) and *N*,*N*-diisopropylethylamine (28 mg, 0.217 mmol) were dissolved in 10 mL of *N*,*N*-dimethylformamide and stirred under nitrogen. Progress of the reaction was monitored by TLC (dichloromethane:acetone, 9.5:0.5, v/v). After 48 h, the crude reaction mixture was separated into two equal portions (to avoid overloading of column) and was purified by size-exclusion chromatography (Bio-Beads S-X1, THF). The initial red elution band corresponding to a higher molecular weight product was collected and further purified by dialysis (3.5 kDa MWCO) against THF:ethanol (1:1, v/v) followed by dichloromethane:ethanol (1:1, v/v). Further purification by dialysis was necessary due to the high tendency of unreacted PDMI-PFP to aggregate with the desired product, causing it to co-elute in the initial red elution band. The dialyzed product was then concentrated in vacuo and further dried under high vacuum to yield PEG_43_-*b*-P(NIPAM_21_-*co*-PDMI_9_) as a dark red solid (123 mg, 74%). *M*_n,NMR_ = 11,000 g mol^−1^; *M*_n,GPC_ = 15,800 g mol^−1^; *Đ* = 1.09.

### Preparation of PEG_43_-*b*-P(NIPAM_21_-*co*-PDMI_9_) self-assemblies

In a typical experiment, a 4-mL sample tube equipped with a magnetic stir bar (10 mm length×5 mm width for total volumes <1.5 mL or 6 mm length×3 mm width for total volumes >1.5 mL) was charged with 0.5 mg of PEG_43_-*b*-P(NIPAM_21_-*co*-PDMI_9_) and the required amount of THF (according to the phase diagram shown in Fig. 3). Gentle shaking was applied to homogenize the solution before required amounts of water was added directly into the polymer solution in a single portion. The mixture was then stirred at room temperature in the fumehood (100 rpm for total volumes <1.5 mL or 135 rpm for total volumes >1.5 mL) to allow the evaporation of THF (~1–3 days, depending on the amount of THF used).

### Data availability

The data that support the findings of this study are available from the corresponding author upon reasonable request.

## Electronic supplementary material

Supplementary Information

## References

[1] Voeltz GK, Prinz WA (2007). Sheets, ribbons and tubules - how organelles get their shape. Nat. Rev. Mol. Cell Biol..

[2] Rangamani P (2013). Decoding information in cell shape. Cell.

[3] Discher DE, Eisenberg A (2002). Polymer vesicles. Science.

[4] Peyret A (2017). Polymersome popping by light-induced osmotic shock under temporal, spatial, and spectral control. Angew. Chem. Int. Ed..

[5] Huang X (2013). Interfacial assembly of protein-polymer nano-conjugates into stimulus-responsive biomimetic protocells. Nat. Commun..

[6] Wong CK (2015). Polymersomes prepared from thermoresponsive fluorescent protein-polymer bioconjugates: capture of and report on drug and protein payloads. Angew. Chem. Int. Ed..

[7] Chandler, D. Interfaces and the driving force of hydrophobic assembly. *Nature* **437**, 640–647 (2005). 10.1038/nature04162 16193038

[8] Hickey RJ (2014). Size-controlled self-assembly of superparamagnetic polymersomes. ACS Nano.

[9] Palivan CG (2016). Bioinspired polymer vesicles and membranes for biological and medical applications. Chem. Soc. Rev..

[10] Deng Z (2016). Engineering intracellular delivery nanocarriers and nanoreactors from oxidation-responsive polymersomes via synchronized bilayer cross-linking and permeabilizing inside live cells. J. Am. Chem. Soc..

[11] Liu J (2016). DNA-mediated self-organization of polymeric nanocompartments leads to interconnected artificial organelles. Nano Lett..

[12] van Oers MCM, Rutjes FPJT, van Hest JCM (2013). Tubular polymersomes: a cross-linker-induced shape transformation. J. Am. Chem. Soc..

[13] Salva R (2013). Polymersome shape transformation at the nanoscale. ACS Nano.

[14] Abdelmohsen LKEA (2016). Formation of well-defined, functional nanotubes via osmotically induced shape transformation of biodegradable polymersomes. J. Am. Chem. Soc..

[15] Rikken RSM (2016). Shaping polymersomes into predictable morphologies via out-of-equilibrium self-assembly. Nat. Commun..

[16] Robertson JD (2014). pH-sensitive tubular polymersomes: formation and applications in cellular delivery. ACS Nano.

[17] Xing X (2012). Morphology of nematic and smectic vesicles. Proc. Natl Acad. Sci. USA.

[18] Vriezema DM (2003). Vesicles and polymerized vesicles from thiophene-containing rod-coil block copolymers. Angew. Chem. Int. Ed..

[19] Warren NJ (2015). Testing the vesicular morphology to destruction: birth and death of diblock copolymer vesicles prepared via polymerization-induced self-assembly. J. Am. Chem. Soc..

[20] Martinez CR, Iverson BL (2012). Rethinking the term ‘pi-stacking’. Chem. Sci.

[21] Hunter CA, Sanders JKM (1990). The nature of π−π interactions. J. Am. Chem. Soc..

[22] Salonen LM, Ellermann M, Diederich F (2011). Aromatic rings in chemical and biological recognition: energetics and structures. Angew. Chem. Int. Ed..

[23] Würthner F (2016). Perylene bisimide dye assemblies as archetype functional supramolecular materials. Chem. Rev..

[24] Lin YL, Chang HY, Sheng YJ, Tsao HK (2012). Photoresponsive polymersomes formed by amphiphilic linear-dendritic block copolymers: generation-dependent aggregation behavior. Macromolecules.

[25] Lin Y-L, Chang H-Y, Sheng Y-J, Tsao H-K (2014). The fusion mechanism of small polymersomes formed by rod–coil diblock copolymers. Soft Matter.

[26] Mondal T, Sakurai T, Yoneda S, Seki S, Ghosh S (2015). Semiconducting nanotubes by intrachain folding following macroscopic assembly of a naphthalene-diimide (NDI) appended polyurethane. Macromolecules.

[27] Roche C (2015). A supramolecular helix that disregards chirality. Nat. Chem..

[28] Wang W (2003). Dynamic π−π stacked molecular assemblies emit from green to red colors. Nano Lett..

[29] Görl D, Zhang X, Würthner F (2012). Molecular assemblies of perylene bisimide dyes in water. Angew. Chem. Int. Ed..

[30] Shao C, Grüne M, Stolte M, Würthner F (2012). Perylene bisimide dimer aggregates: fundamental insights into self-assembly by NMR and UV/vis spectroscopy. Chemistry.

[31] Echue G, Lloyd-Jones GC, Faul CFJ (2015). Chiral perylene diimides: building blocks for ionic self-assembly. Chemistry.

[32] Rehm S, Stepanenko V, Zhang X, Rehm TH, Würthner F (2010). Spermine-functionalized perylene bisimide dyes-highly fluorescent bola-amphiphiles in water. Chemistry.

[33] Fennel F (2013). Biphasic self-assembly pathways and size-dependent photophysical properties of perylene bisimide dye aggregates. J. Am. Chem. Soc..

[34] Feil H, Bae YH, Feijen J, Kim SW (1993). Effect of comonomer hydrophilicity and ionization on the lower critical solution temperature of *N*-isopropylacrylamide copolymers. Macromolecules.

[35] Yin X, Hoffman AS, Stayton PS (2006). Poly(*N*-isopropylacrylamide-*co*-propylacrylic acid) copolymers that respond sharply to temperature and pH. Biomacromolecules.

[36] Kotsuchibashi Y, Ebara M, Yamamoto K, Aoyagi T (2011). Tunable stimuli-responsive self-assembly system that forms and stabilizes nanoparticles by simple mixing and heating/cooling of selected block copolymers. Polym. Chem..

[37] Marsden HR, Gabrielli L, Kros A (2010). Rapid preparation of polymersomes by a water addition/solvent evaporation method. Polym. Chem..

[38] Schneider CA, Rasband WS, Eliceiri KW (2012). NIH Image to ImageJ: 25 years of image analysis. Nat. Methods.

[39] Danino D (2012). Cryo-TEM of soft molecular assemblies. Curr. Opin. Colloid Interface Sci..

[40] Yu S, Azzam T, Rouiller I, Eisenberg A (2009). ‘Breathing’ vesicles. J. Am. Chem. Soc..

[41] Sun H (2015). The role of capsule stiffness on cellular processing. Chem. Sci..

[42] Battaglia G, Ryan AJ (2005). Bilayers and interdigitation in block copolymer vesicles. J. Am. Chem. Soc..

[43] Zhu Y, Fan L, Yang B, Du J (2014). Multifunctional homopolymer vesicles for facile immobilization of gold nanoparticles and effective water remediation. ACS Nano.

[44] Zhang K, Jiang M, Chen D (2012). DNA/polymeric micelle self-assembly mimicking chromatin compaction. Angew. Chem. Int. Ed..

[45] Weirich KL (2017). Liquid behavior of cross-linked actin bundles. Proc. Natl Acad. Sci. USA.

[46] Zhu J (2013). Disk-cylinder and disk-sphere nanoparticles via a block copolymer blend solution construction. Nat. Commun..

[47] Martens S, McMahon HT (2008). Mechanisms of membrane fusion: disparate players and common principles. Nat. Rev. Mol. Cell Biol..

[48] McMahon HT, Kozlov MM, Martens S (2010). Membrane curvature in synaptic vesicle fusion and beyond. Cell.

[49] Zhang R (2016). Controlled deformation of vesicles by flexible structured media. *Sci*. Adv..

[50] Struijk CW (2000). Liquid crystalline perylene diimides: architecture and charge carrier mobilities. J. Am. Chem. Soc..

[51] Wicklein A, Lang A, Muth M, Thelakkat M (2009). Swallow-tail substituted liquid crystalline perylene bisimides: synthesis and thermotropic properties. J. Am. Chem. Soc..

[52] Muth MA, Carrasco-Orozco M, Thelakkat M (2011). Liquid-crystalline perylene diester polymers with tunable charge-carrier mobility. Adv. Funct. Mater..

[53] Dubois M (2001). Self-assembly of regular hollow icosahedra in salt-free catanionic solutions. Nature..

[54] Leung CY (2012). Molecular crystallization controlled by pH regulates mesoscopic membrane morphology. ACS Nano.

[55] Gröschel AH (2012). Precise hierarchical self-assembly of multicompartment micelles. Nat. Commun..

[56] Löbling TI (2016). Rational design of ABC triblock terpolymer solution nanostructures with controlled patch morphology. Nat. Commun..

[57] Dasgupta S, Auth T, Gompper G (2014). Shape and orientation matter for the cellular uptake of nonspherical particles. Nano Lett..

