# Supplementary material for: Formation of non-spherical polymersomes driven by hydrophobic directional aromatic perylene interactions
Source: Nat Commun. 2017 Nov 1;8:1240. doi: 10.1038/s41467-017-01372-z (PMC5665895; doi:10.1038/s41467-017-01372-z)
Supplement: Supplementary file 1 — Supplementary Information [file 41467_2017_1372_MOESM1_ESM.pdf]

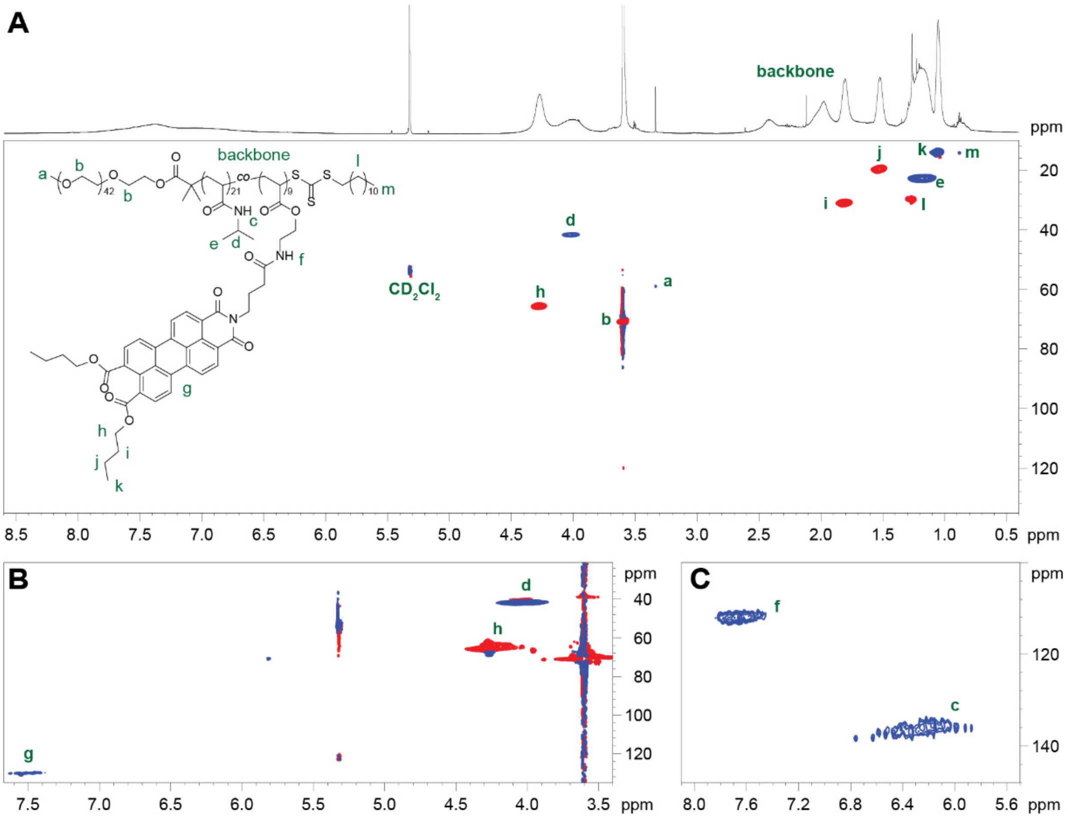

Supplementary Figure 1. (A) ^{1}H-^{13}C HSQC NMR spectrum of PEG_{43}-*b*-P($NIPAM_{21}$-*co*-$PDMI_9$) in CD_2Cl_2 showing strong cross-peaks corresponding to key segments of the diblock terpolymer. (B) Expanded region of the ^{1}H-^{13}C HSQC NMR spectrum above showing weaker cross-peaks corresponding to the aromatic protons. (C) ^{1}H-^{15}N HSQC NMR spectrum of PEG_{43}-*b*-P($NIPAM_{21}$-*co*-$PDMI_9$) in CD_2Cl_2 showing cross-peaks corresponding to the two different amide protons of the diblock terpolymer. Positive and negative contours for both spectra are shown in blue and red, respectively.

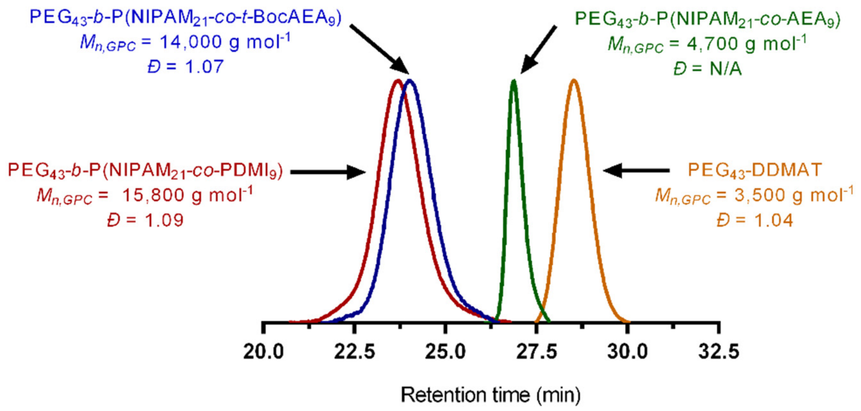

Supplementary Figure 2. DMAc GPC traces of PEG_{43}-DDMAT, PEG_{43}-*b*-P($NIPAM_{21}$-*co*-*t*-$BocAEA_9$), PEG_{43}-*b*-P($NIPAM_{21}$-*co*-AEA_9) and PEG_{43}-*b*-P($NIPAM_{21}$-*co*-$PDMI_9$). Note that the GPC analysis provided an unreliable dispersity (*Đ*) value of 1.01 for PEG_{43}-*b*-P($NIPAM_{21}$-*co*-AEA_9).

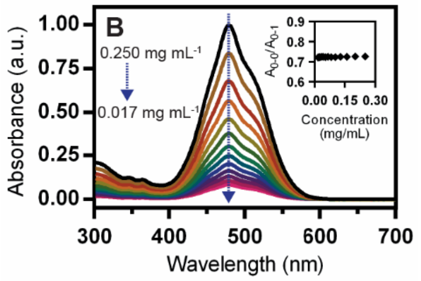

Supplementary Figure 3. Concentration-dependent absorption spectra of PEG_{43}-*b*-P($NIPAM_{21}$-*co*-$PDMI_9$) showing little to no changes in the $A_{0\text{-}0}/A_{0\text{-}1}$ values (inset) even at very low concentrations (0.017 mg mL^{-1}) in tetrahydrofuran, indicating that there are no significant interchain interactions but very strong intrachain interactions between the perylene units on the diblock terpolymer.

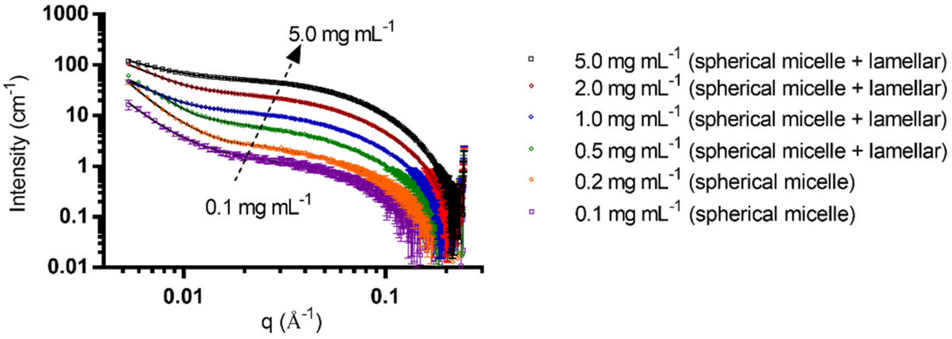

Supplementary Figure 4. SAXS patterns obtained for PEG$_{43}$-*b*-P(NIPAM$_{21}$-*co*-PDMI$_{9}$) at different concentrations in tetrahydrofuran. Solid black lines show fits to the data using either a spherical micelle model or a sum model derived using a linear combination of spherical micelle model and lamellar model.

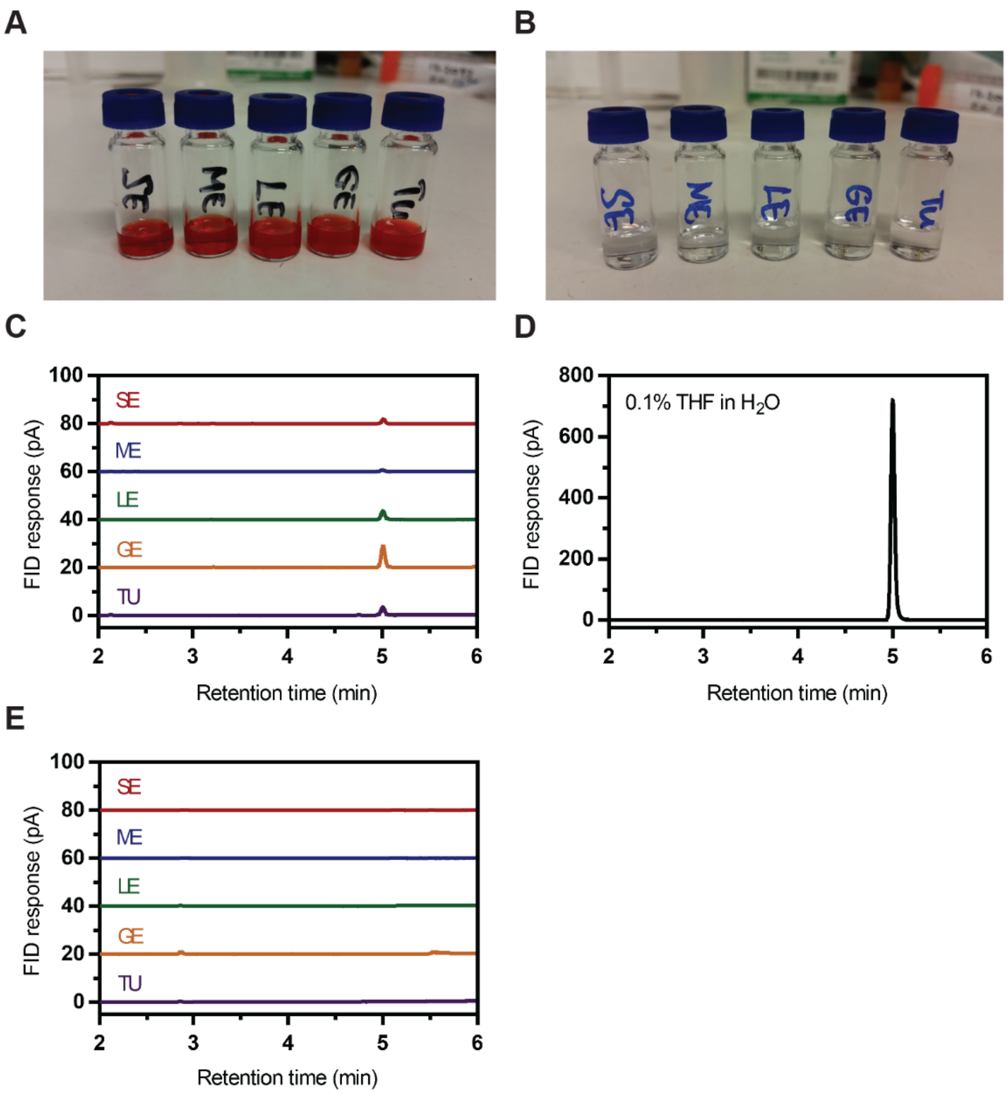

Supplementary Figure 5. Photographs of PEG43-*b*-P(NIPAM21-*co*-PDMI9) micelle/polymersome solutions (A) before and (B) after filtration through 0.2 μm Millex-GN nylon membrane syringe filter units. (C) Gas chromatography-flame ionization detector (GC-FID) analysis of filtered micelle/polymersome solutions (plots are offset for clarity). The peaks at 5 min corresponds to residual THF left in each sample after evaporation under stirring for at least 72 h in the fumehood. (D) GC-FID trace of 0.1% (v/v) THF in water for comparison. (E) GC-FID trace of filtered micelle/polymersome solutions after extensive dialysis (2 days) against water using 3.5 kDa MWCO dialysis tubing.

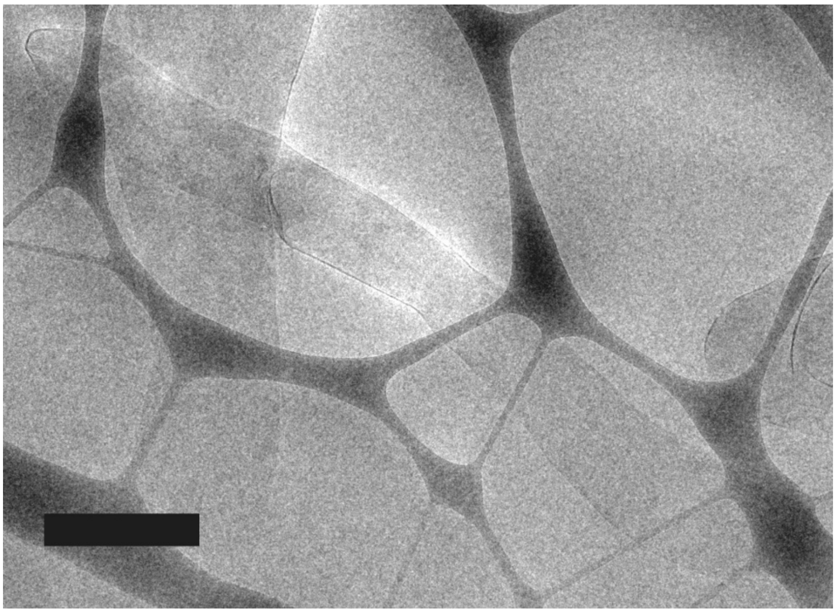

Supplementary Figure 6. Enlarged region of the same cryo-TEM image of TU shown in Fig. 2j in the main text. Scale bar, 1 μm.

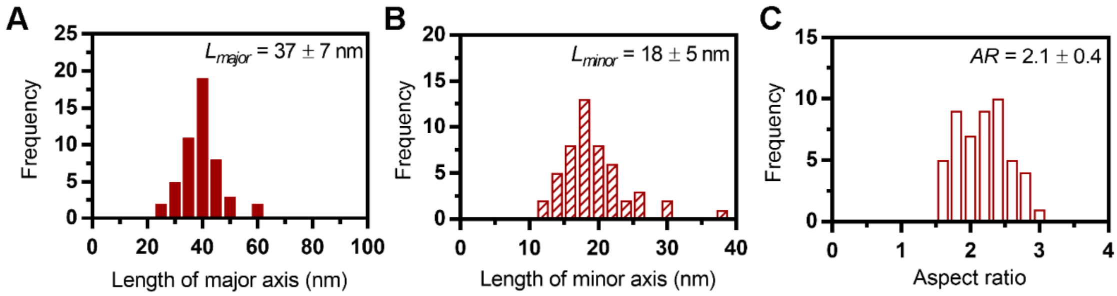

Supplementary Figure 7. Representative histograms for the (A) major axis lengths, (B) minor axis lengths and (C) aspect ratios of small ellipsoidal micelles (SE).

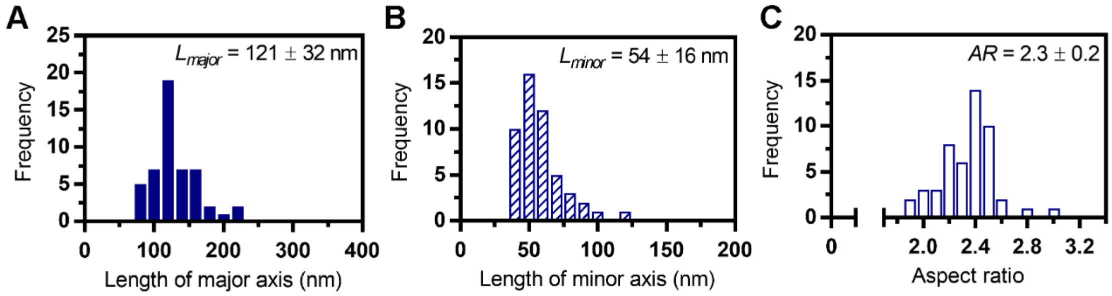

Supplementary Figure 8. Representative histograms for the (A) major axis lengths, (B) minor axis lengths and (C) aspect ratios of medium ellipsoidal polymersomes (ME).

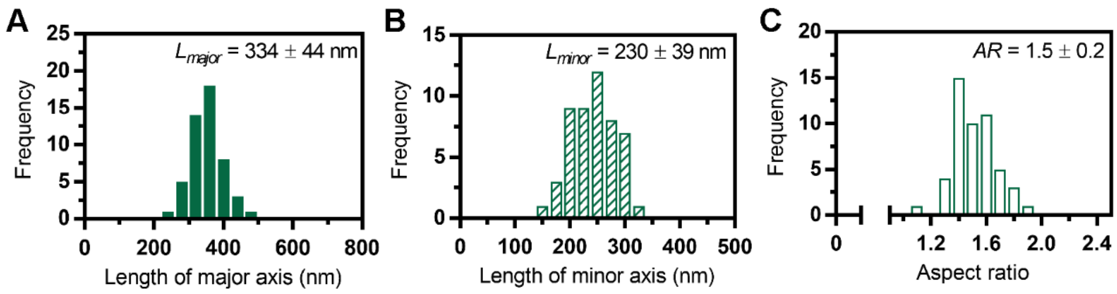

Supplementary Figure 9. Representative histograms for the (A) major axis lengths, (B) minor axis lengths and (C) aspect ratios of large ellipsoidal polymersomes (LE).

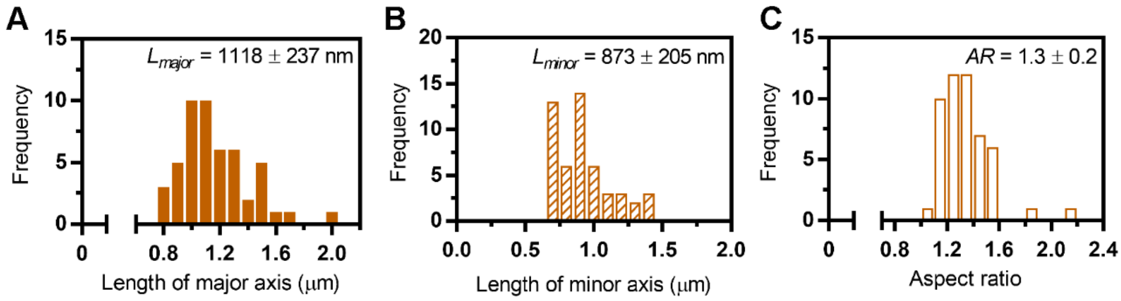

Supplementary Figure 10. Representative histograms for the (A) major axis lengths, (B) minor axis lengths and (C) aspect ratios of giant ellipsoidal polymersomes (GE).

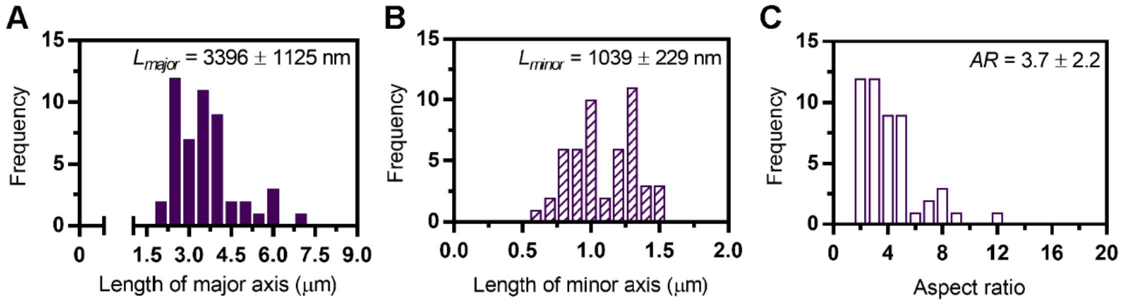

Supplementary Figure 11. Representative histograms for the (A) major axis lengths, (B) minor axis lengths and (C) aspect ratios of tubular polymersomes (TU).

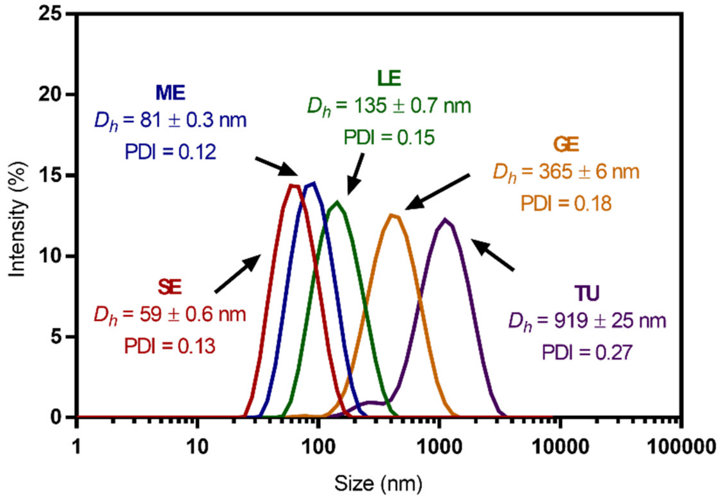

Supplementary Figure 12. Particle size distribution of all five morphologies determined by dynamic light scattering (DLS) measurements. D_h = Z-average hydrodynamic diameter.

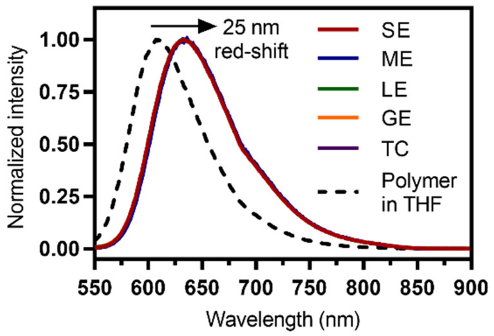

Supplementary Figure 13. Normalized fluorescence spectra of all five morphologies observed.

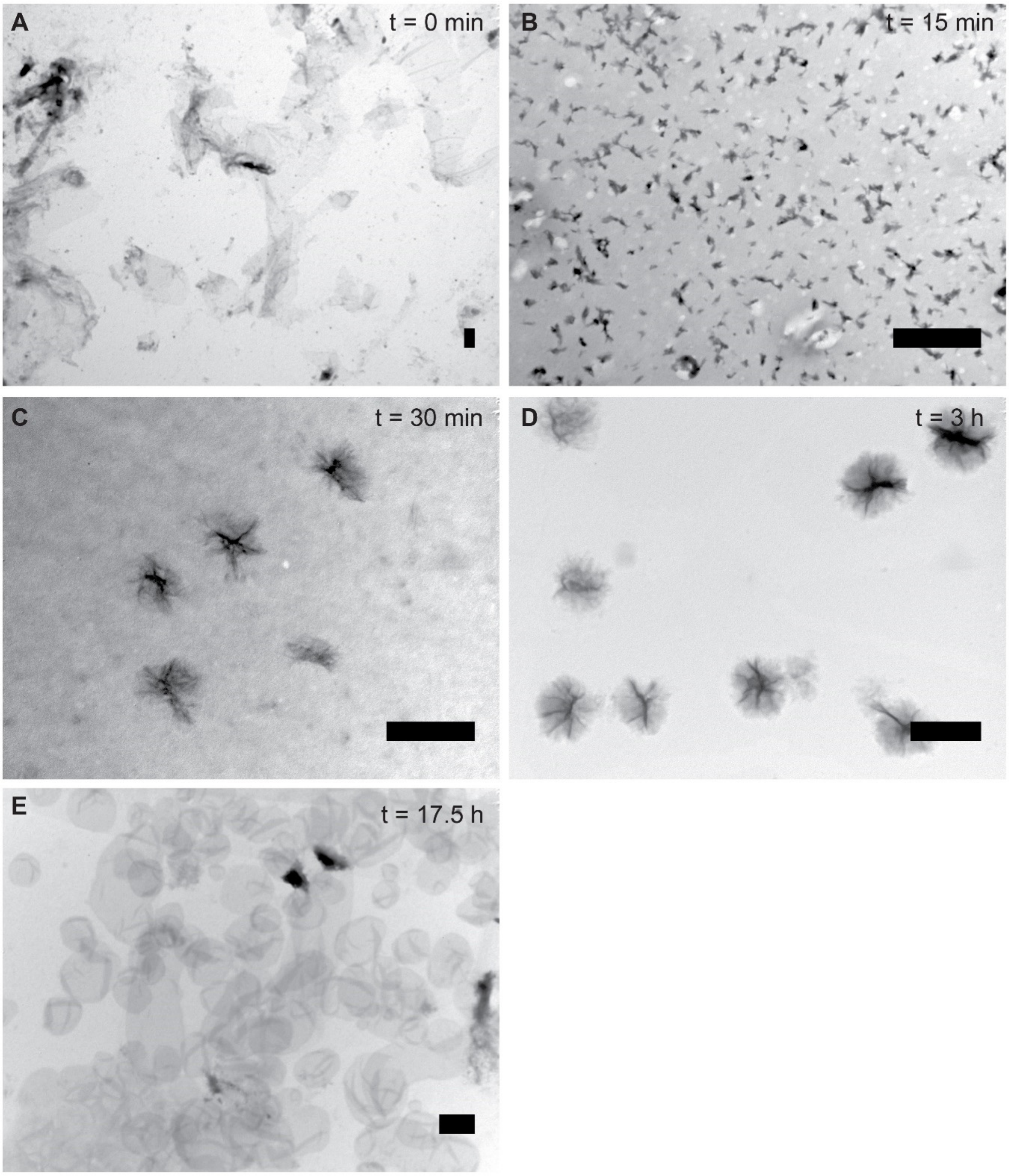

Supplementary Figure 14. Intermediate structures observed at different time points during the self-assembly process of tubular polymersomes (TU). (A) 0 min, (B) 15 min, (C) 30 min, (D) 3 h, (E) 17.5 h. Scale bar, 1 μm.

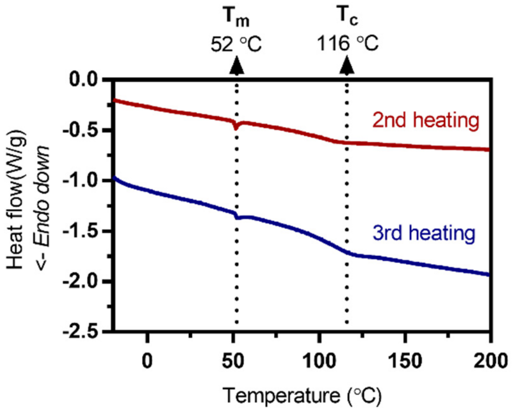

Supplementary Figure 15. Differential scanning calorimetry (DSC) curves of PEG_{43}-*b*-P($NIPAM_{21}$-*co*-$PDMI_9$) during the second and third heating run. The first heating run (not shown) was performed to erase previous thermal history of the polymer. A heating rate of 5 °C min^{-1} was used.

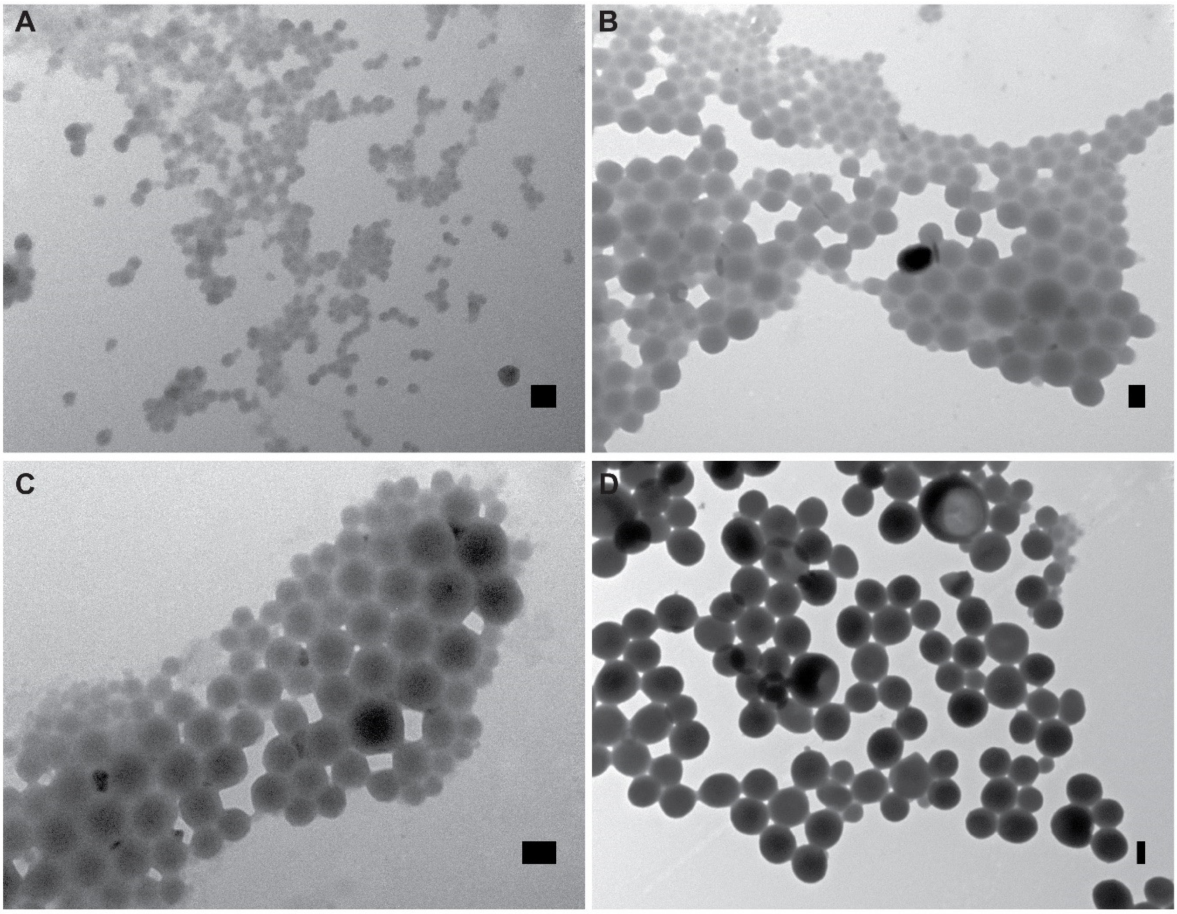

Supplementary Figure 16. TEM images solutions of (A) ME, (B) LE, (C) GE and (D) TU after annealing at 85 °C for 90 min in the presence of 35 μL of THF. These spherical morphologies are likely equilibrium shapes for our system since they do not revert back to their original non-spherical shapes even upon slow cooling to room temperature after annealing. Scale bar, 100 nm.

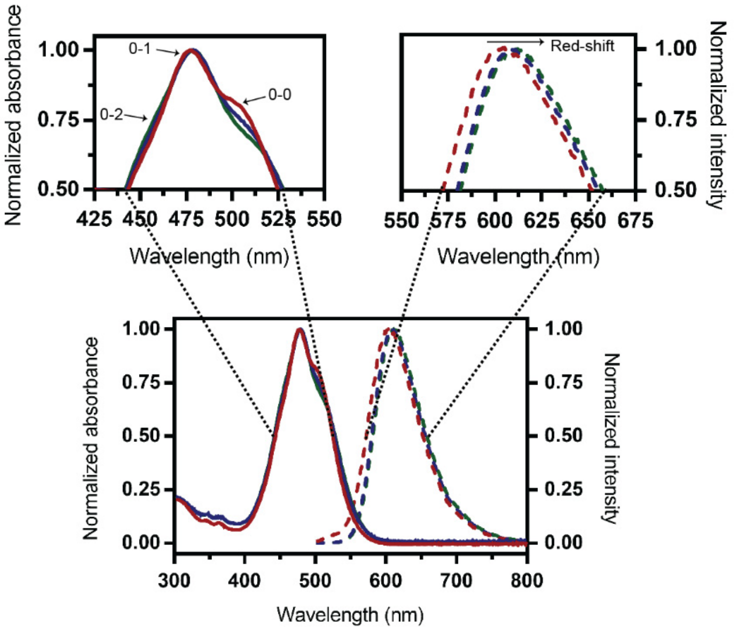

Supplementary Figure 17. Normalized absorption (solid lines) and fluorescence spectra (dashed lines) of PEG$_{43}$-*b*-P(NIPAM$_{22}$-*co*-PDMI$_5$) (red), PEG$_{43}$-*b*-P(NIPAM$_{21}$-*co*-PDMI$_9$) (blue) and PEG$_{43}$-*b*-P(NIPAM$_{23}$-*co*-PDMI$_{19}$) (green) in THF. Enlarged regions of the same spectra are shown above for clarity. The decrease in 0-0 transition in the absorption spectra and red-shift observed in the emission spectra as perylene content increases indicates that aromatic stacking strength increases in the order of PEG$_{43}$-*b*-P(NIPAM$_{22}$-*co*-PDMI$_5$) > PEG$_{43}$-*b*-P(NIPAM$_{21}$-*co*-PDMI$_9$) > PEG$_{43}$-*b*-P(NIPAM$_{23}$-*co*-PDMI$_{19}$).

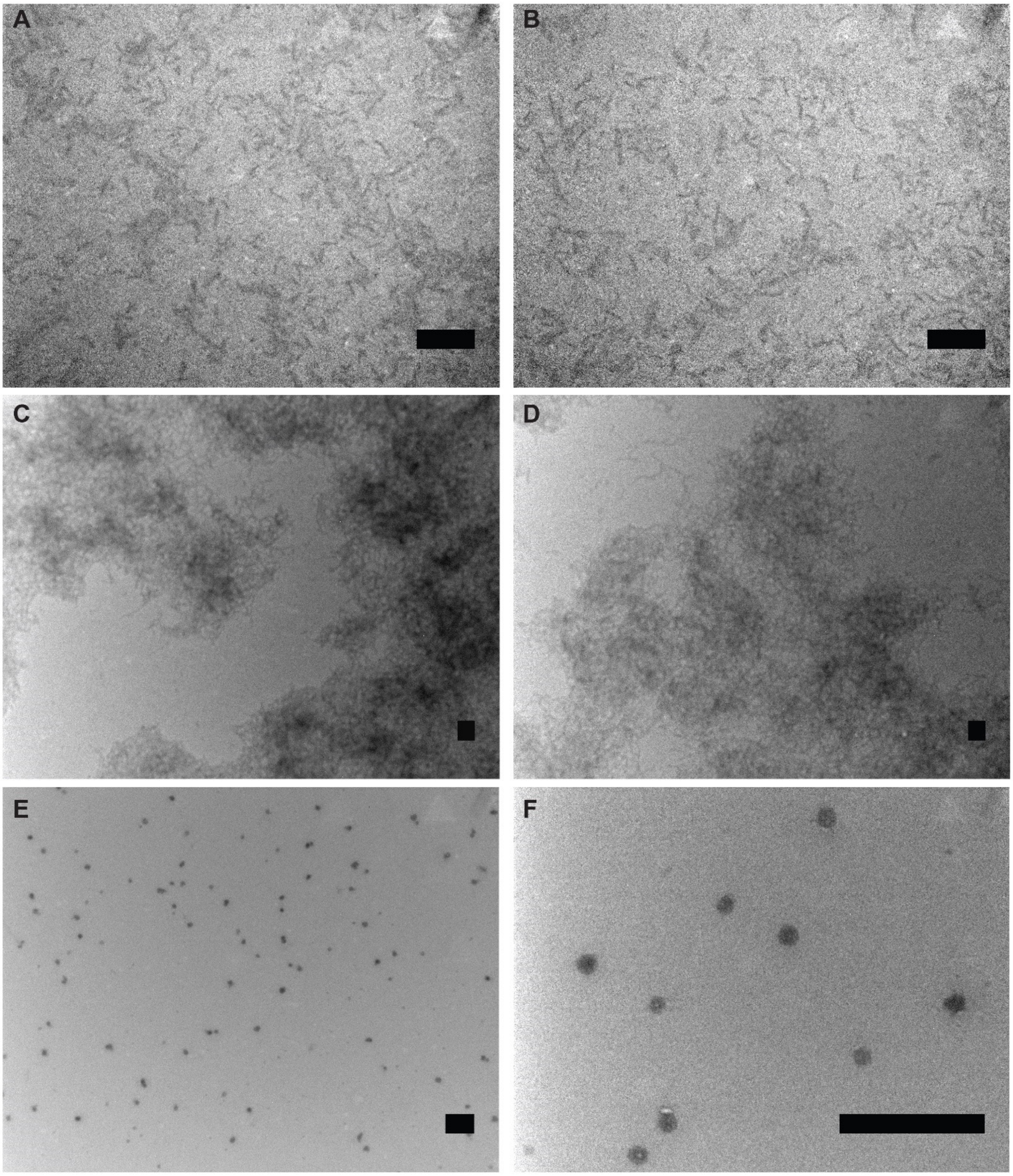

Supplementary Figure 18. Self-assemblies formed by PEG_{43}-*b*-P($NIPAM_{22}$-*co*-$PDMI_5$) using the 'solvent-switch' method. (A, B) Worm-like micelles prepared with an initial polymer concentration in THF = 0.83 mg mL^{-1}. (C, D) Longer worm-like micelles prepared with an initial polymer concentration in THF = 0.50 mg mL^{-1}. (E, F) Spherical micelles prepared with an initial polymer concentration in THF = 0.25 mg mL^{-1}. In all cases, the final polymer concentration in water = 1.00 mg mL^{-1}. Scale bar, 200 nm.

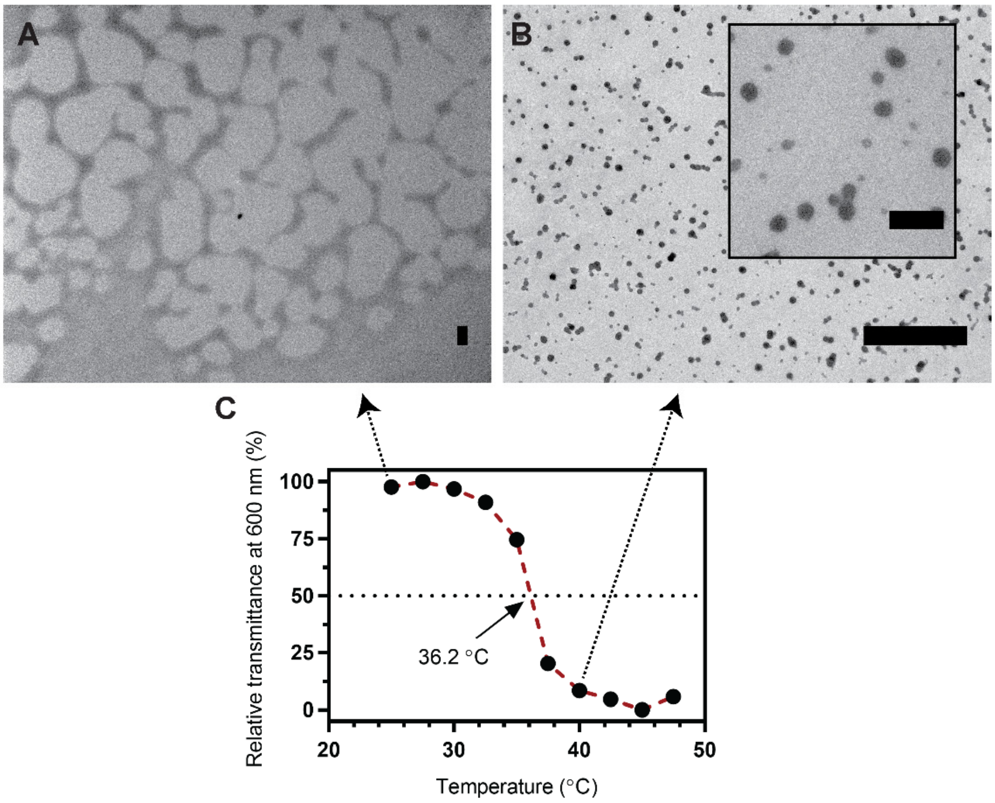

Supplementary Figure 19. TEM images of (A) undefined (dewetted) structures formed by PEG_{43}-*b*-P($NIPAM_{21}$-*co*-*t*-$BocAEA_9$) after subjection to the "solvent-switch" process and (B) spherical micellar-like structures formed by PEG_{43}-*b*-P($NIPAM_{21}$-*co*-*t*-$BocAEA_9$) after subjection to the "solvent-switch" process and incubation at 40 °C (a higher magnification image is shown inset). (C) Cloud point profile of PEG_{43}-*b*-P($NIPAM_{21}$-*co*-*t*-$BocAEA_9$). The cloud point or estimated LCST of PEG_{43}-*b*-P($NIPAM_{21}$-*co*-*t*-$BocAEA_9$), defined as the temperature that results in a relative transmittance of 50%, was determined to be 36.2 °C. In summary, these results above illustrate the importance of having aromatic perylenes on the polymer for non-spherical polymersome formation. Scale bars, 1 µm and 200 nm inset.

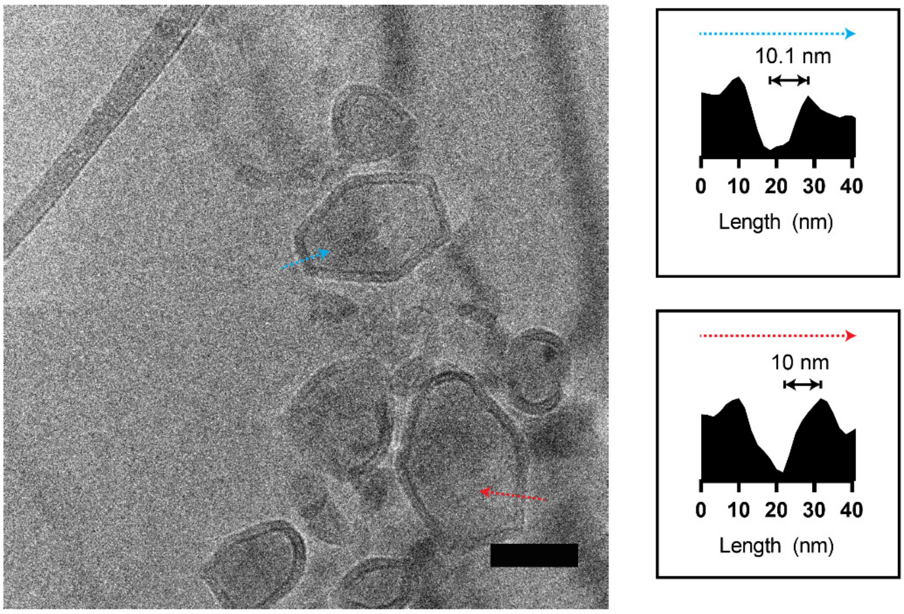

Supplementary Figure 20. Cryo-TEM image of polyhedral (faceted) polymersomes prepared from PEG_{43}-*b*-P($NIPAM_{23}$-*co*-$PDMI_{19}$) using the "solvent-switch" method. The estimated membrane thickness of ~10.1 nm suggests that these polymersomes have an interdigitated bilayer membrane structure too. Scale bar, 100 nm.

Supplementary Table 1. Integration values and relative integration values for ^{1}H-^{15}N cross-peaks **f** and **c** shown in Supplementary Fig. 1. The degree of dye functionalization on the diblock terpolymer was obtained by multiplying the known number of repeating units of NIPAM (i.e. 21 repeat units, determined using integrals from the ^{1}H NMR spectrum (Supplementary Fig. 37) of PEG-*b*-P(NIPAM$_{21}$-*co*-AEA$_{9}$) by the relative integration value of 0.42 (*i.e.* 21 × 0.42 = 8.82 ≈ 9 repeat units of PDMI).

Peak	Integral (abs)	Integral (rel)	ν(F2) (ppm)	ν(F1) [ppm]
**f**	8.80E+06	0.42	7.56	111.8
**c**	2.11E+07	1.00	6.16	137.3

Supplementary Table 2. Integrated peak areas and relative peak areas under the GC-FID peaks shown in Supplementary Fig. 5C used to estimate the amount of residual THF left in the micelle/polymersome solutions after evaporation under stirring in the fumehood for 72 h. Standard = 0.1% (v/v) THF in water.

Sample	Peak area (counts)	Peak area (rel.)	%THF in H_2O	V_{THF} in 1 mL H_2O (nL)	V_{THF} used to prepare particles (µL)	%THF (v/v) left
**SE**	5.2	402	0.0002	2.5	100	0.0025
**ME**	2.0	1058	0.0001	0.9	600	0.0002
**LE**	10.5	201	0.0005	5.0	800	0.0006
**GE**	25.1	84	0.0012	11.9	1000	0.0012
**TU**	10.1	207	0.0005	4.8	2000	0.0002
**Standard**	2103.9	1	0.1000	1000	-	-

Supplementary Methods

Materials and instrumentation

All chemicals were purchased from commercial sources and were used as received unless otherwise stated. *N*-isopropylacrylamide was recrystallized from hexane three times prior to use. Deuterated chloroform was filtered over anhydrous potassium carbonate and neutral aluminium oxide prior to use.

Gel Permeation Chromatography (GPC) analyses were performed on a Shimadzu modular system, comprised of a DGU-12A degasser, an LC-10AT pump, an SIL-10AD auto-injector, a CTO-10A column oven and an RID-10A refractive index detector. The system is equipped with a 50 x 7.8 mm guard column and four 300 x 7.8 mm linear columns (500, 10^3, 10^4, 10^5 Å pore size, 5 μm particle size). *N*,*N*-dimethylacetamide (DMAc, HPLC grade, 0.05% w/v BHT, 0.03% w/v LiBR) was used as the eluent at a flow rate of 1 mL min^{-1}. The system was calibrated with poly(methyl methacrylate) (PMMA) standards ranging from 500 to 10^6 g mol^{-1}. All samples were filtered with 0.22 μm syringe filters prior to analysis.

Nuclear magnetic resonance (NMR) spectroscopy measurements were recorded on either a Bruker Avance III 300 MHz NMR spectrometer, Bruker Avance III 400 MHz NMR spectrometer (for 1D NMRs; ^{1}H, ^{13}C and ^{19}F) or a Bruker Avance III 600 MHz NMR spectrometer equipped with a 5 mm TCl cryoprobe (for all two-dimensional NMRs; ^{1}H-^{13}C and ^{1}H-^{15}N HSQC). All spectra were recorded at 298 K (with the exception of PEG_{43}-*b*-$P(NIPAM_{23}$-*co*-$PDMI_{19})$ which was measured at 343 K). Chemical shifts are expressed in parts per million (ppm) and referenced to residual deuterated solvent peaks. All NMR data were acquired and processed using either TopSpin 3.1 or 3.5 software (Bruker). Abbreviations used

to describe multiplicities are reported as follows: s, singlet; d, doublet; t, triplet; m, multiplet; dd, doublet of doublets; br s, broad singlet.

UV-Vis spectroscopy and fluorescence spectroscopy measurements were recorded using a Varian Cary 50 Bio UV-Vis spectrophotometer and a Varian Cary Eclipse fluorescence spectrophotometer, respectively. Absorption and fluorescence spectra of samples were recorded in a 0.7 mL dual pathlength (1.0/0.2 cm) quartz cuvette.

Transmission electron microscopy (TEM) analyses were performed on a JEOL1400 TEM operating at an accelerating voltage of 100 kV. Samples were prepared by dropping 10 μL of 0.1 mg mL^{-1} polymer solution onto a formvar/carbon-coated copper grid (n.b. a carbon only-coated copper grid was used for the analysis of 0.1 mg mL^{-1} PEG_{43}-*b*-P($NIPAM_{21}$-*co*-$PDMI_9$) in tetrahydrofuran (refer to Fig. 1c) due to the incompatibility of formvar with tetrahydrofuran). The droplet was left on the grid for at least 2 minutes before being blotted off with a filter paper. The grid was then left to air-dry overnight prior to analysis. No staining was used. TEM images were analyzed using ImageJ for the determination of minor/major axis lengths (ellipsoidal particles were measured using an elliptical region of interest (ROI) selection tool; tubular structures were measured using a segmented line tool) and membrane thicknesses of the self-assemblies obtained.

Cryogenic-transmission electron microscopy (cryo-TEM) analyses were performed on an FEI Tecnai G2 TEM operating at an accelerating voltage of 200 kV. Images were acquired using a BM Eagle 2K CCD Camera and an in-built low-dose software. Samples were vitrified using a Leica EM GP vitrification robot following a general procedure, as follows: 6 μL of aqueous

0.5 mg mL^{-1} PEG_{43}-*b*-P($NIPAM_{21}$-*co*-$PDMI_9$) solution was pipetted onto a 300 mesh copper grid with lacey formvar/carbon film support. The sample droplet was allowed to equilibrate for 30 seconds at room temperature and at 90% relative humidity, before being blotted from one side for 1.8 seconds. The blotted grid was subsequently plunged into liquid ethane held at ~-174 °C. Excess ethane was blotted away with a piece of pre-cooled filter paper. The vitrified grid was stored in a cryo grid box immersed in liquid nitrogen before finally being cryo-transferred into the microscope using a Gatan 626 cryo-transfer holder.

Confocal laser-scanning microscopy (CLSM) analyses were performed on a Leica TCS SP5, equipped with a 63.0x1.40NA oil immersion objective. Fluorescence images were recorded (HyD detector, spectral window of 500-630 nm) using an Ar laser excitation at 488 nm. Z-stacks were acquired in 256 x 256 pixels resolution and in 0.2 µm step sizes (42 images in total, 8.6 µm *z*-height). All samples were embedded in agarose gel prior to imaging to eliminate polymersome motion in solution. For a typical sample preparation, a 1.5% agarose solution was first heated to 70 °C to aid dissolution before being allowed to cool to approximately 45 °C. 50 µL of the partially-cooled agarose solution was then quickly mixed with 50 µL of 0.15 mg mL^{-1} PEG_{43}-*b*-P($NIPAM_{21}$-*co*-$PDMI_9$) polymersome solution and transferred onto a glass bottom petri dish (Fluorodish). The mixture/gel was allowed to sit for 30 min before imaging.

Synchrotron small-angle X-ray scattering (SAXS) measurements were performed on the SAXS/WAXS beamline at the Australian Synchrotron facility at a photon energy of 12 keV (1.0322 Å wavelength). The sample-to-detector distance was calibrated to 3.344 m to give a q-range of 0.005 – 0.25 $Å^{-1}$. The scattering vector, q follows the equation $q = (4\pi sin\theta)/\lambda$ where 2θ is the scattering angle and λ is the incident X-ray wavelength. Static sample

measurements were performed in 0.01 mm thin-walled quartz capillary tubes. Data acquisition and reduction were carried out using the scatterBrain software developed by the Australian Synchrotron. Data fittings were performed using the SasView software obtained from http://www.sasview.org/.

Differential scanning calorimetry (DSC) measurement was carried out on a TA Instruments DSC Q20 equipped with an RCS40 refrigerated cooling system. 4.8 mg of sample was weighed in an aluminium pan and cycled between -30 °C to 210 °C at a heating/cooling rate of 5 °C min^{-1}.

Dynamic light scattering (DLS) measurements were performed on a Zetasizer Nano ZSP equipped with optical filters for improved measurement of fluorescent samples. Samples were prepared at a concentration of 0.1 mg mL^{-1} and measured in disposable cuvettes at 25 °C, using a backscatter angle of 173°.

Synthesis and characterization of PDMI-PFP

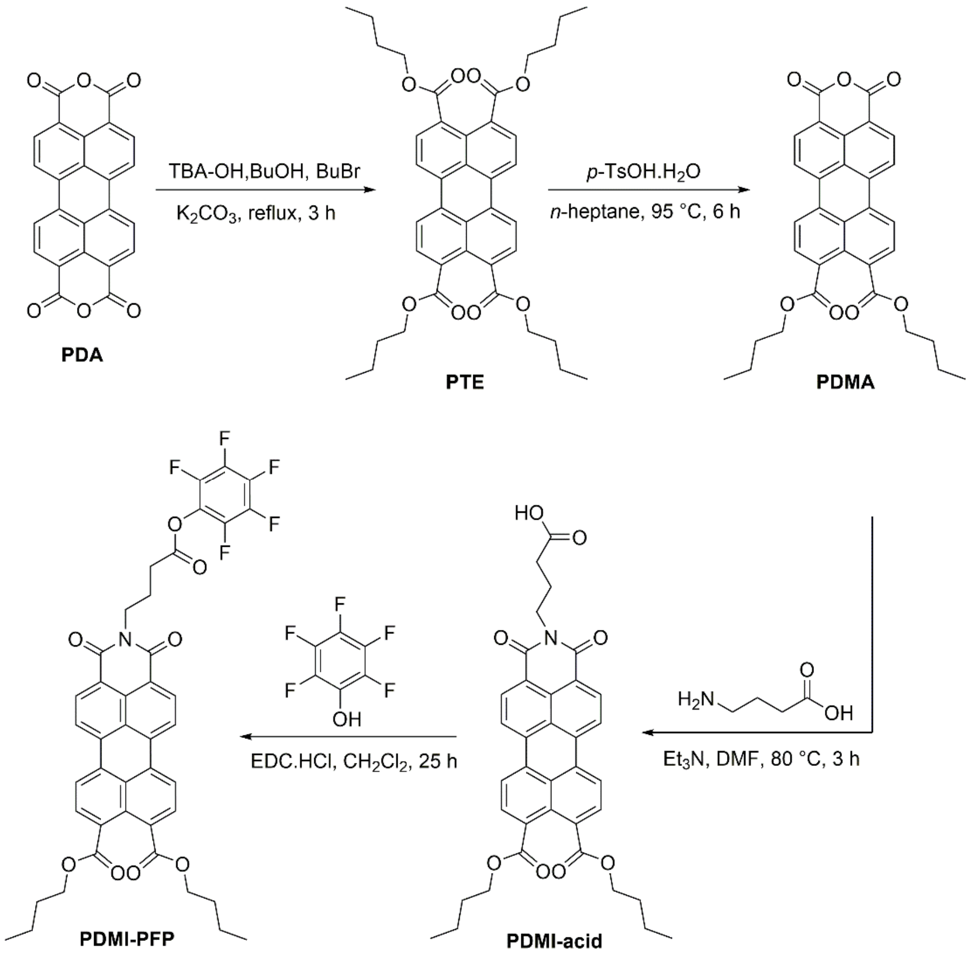

Supplementary Figure 21. Synthetic route to obtain the amine-reactive perylene diester monoimide derivative PDMI-PFP.

Synthesis of perylene-3,4,9,10-tetracarboxylate tetrabutyl ester (PTE)

A 250 mL round-bottomed flask was charged with perylene-3,4,9,10-tetracarboxylic dianhydride (PDA) (10 g, 25.5 mmol), tetrabutylammonium hydroxide (30 mL, 0.12 mol) and 1-butanol (40 mL). The mixture was sonicated to aid the dissolution of PDA. After PDA was completely dissolved, 1-bromobutane (40 mL, 0.37 mol) and potassium carbonate (10 g, 72 mmol) was added and the mixture was refluxed for 3 h. The crude reaction mixture was allowed to cool to room temperature. The yellow precipitate in the cooled mixture was collected by

suction filtration and washed with copious amounts of methanol and water. The yellow precipitate was then redissolved in 100 mL of chloroform and dried over anhydrous magnesium sulphate before being passed through a silica plug, using chloroform as the eluent. The filtered solution was then concentrated *in vacuo* and the resulting yellow solid obtained was air-dried at room temperature overnight and further dried under high vacuum to yield *perylene-3,4,9,10-tetracarboxylate tetrabutyl ester* (PTE) as a yellow solid (15.8 g, 95%). ^{1}H NMR (300 MHz, $CDCl_3$) δ (ppm): 8.16 (d, J = 8.0 Hz, 4H, H_{Ar}), 7.97 (d, J = 7.9 Hz, 4H, H_{Ar}), 4.34 (t, J = 6.8 Hz, 8H, -COOCH_2-), 1.79 (m, 8H, -$COOCH_2$-CH_2-), 1.50 (m, 8H, -CH_2-CH_3), 1.00 (t, J = 7.4, 12H, -CH_2-CH_3). ^{13}C NMR (75 MHz, $CDCl_3$) δ (ppm): 168.7, 133.0, 130.5, 129.0, 128.8, 121.4, 65.5, 30.8, 19.4, 14.0. MS (MALDI-TOF) m/z = 653.36 $[M+H]^+$, calcd. for $C_{40}H_{45}O_8$ = 653.31. These results are in good agreement with those reported in literature.[1]

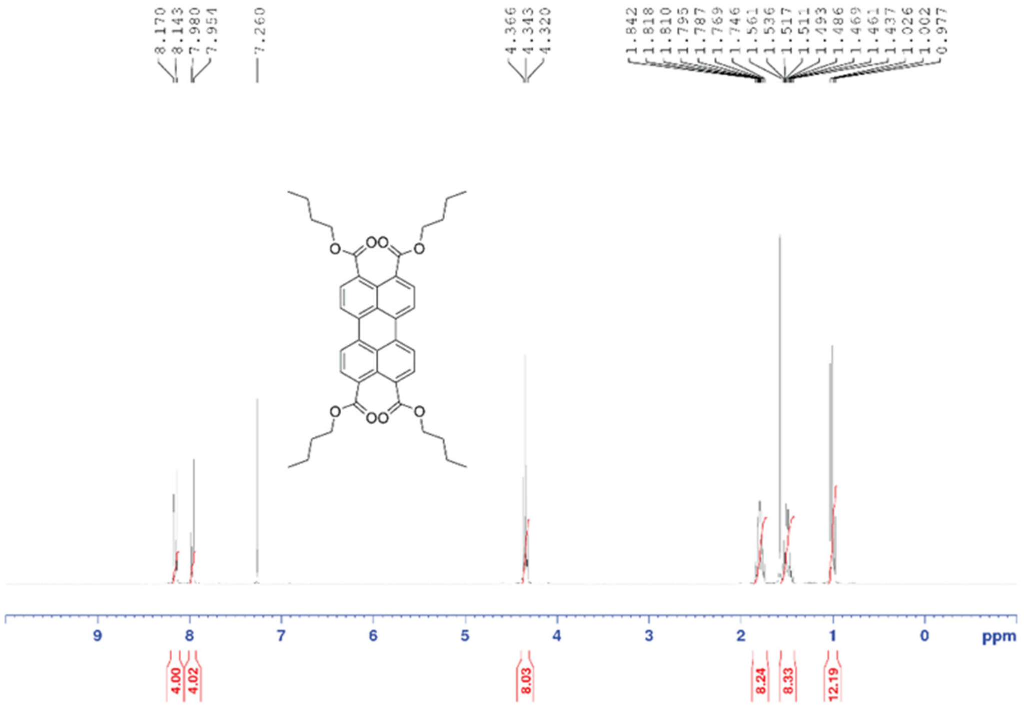

Supplementary Figure 22. ^{1}H NMR spectrum of PTE in $CDCl_3$.

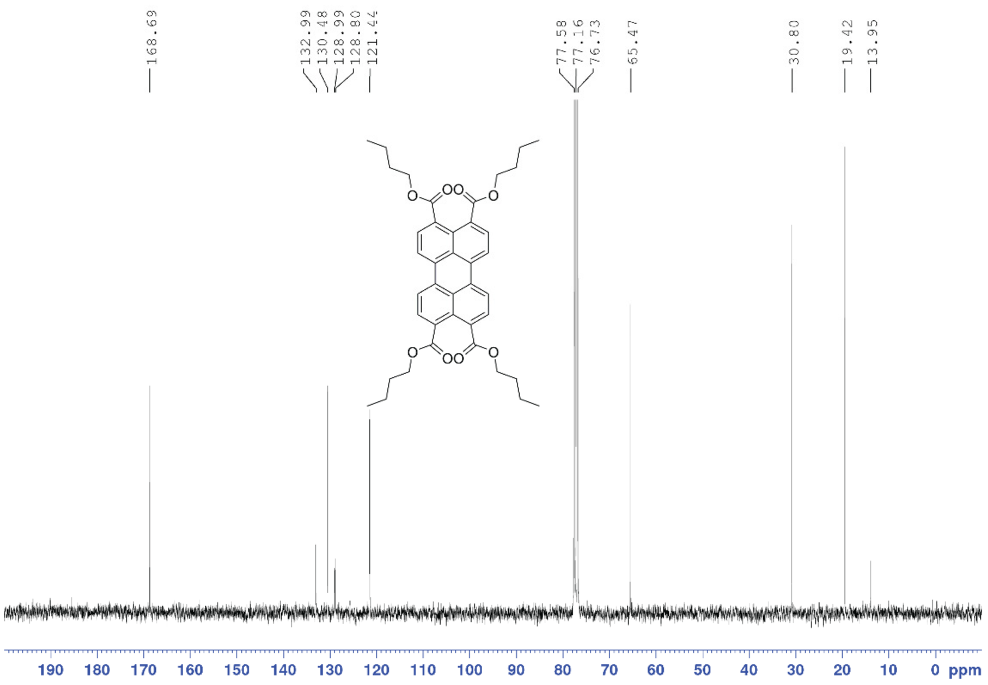

Supplementary Figure 23. ^{13}C NMR spectrum of PTE in $CDCl_3$.

Synthesis of perylene-3,4,9,10-tetracarboxylic dibutyl ester monoanhydride (PDMA)

This compound was synthesized following a slightly modified literature procedure.[2] A 25 mL round-bottomed flask was charged with perylene-3,4,9,10-tetracarboxylate tetrabutyl ester (PTE) (2.01 g, 3.08 mmol) and 6 mL of toluene/*n*-heptane (1:5 v/v) mixture. The solution was stirred at 95 °C to aid the dissolution of PTE. After PTE was completely dissolved, *p*-toluenesulphonic acid monohydrate (612 mg, 3.22 mmol) was added and the mixture was further stirred at the same temperature, during which the formation of a dark red precipitate was observed. Progress of the reaction was monitored by TLC (dichloromethane:methanol, 9:1, v/v). After 6 h, the crude reaction mixture was allowed to cool to room temperature. The dark red precipitate in the cooled mixture was collected by suction filtration, washed with copious amounts of water and methanol, air-dried at room temperature overnight and further

dried under high vacuum to yield *perylene-3,4,9,10-tetracarboxylic dibutyl ester monoanhydride* (PDMA) as a dark red solid (1.55 g, 96%). The resulting product contained small amounts of unreacted PTE impurities but was used in the next step without further any purification due to solubility limitations. ^{1}H NMR (300 MHz, $CDCl_3$) δ (ppm): 8.64 (d, J = 8.0 Hz, 2H, H_{Ar}), 8.51 (m, 4H, H_{Ar}), 8.13 (d, J = 7.9 Hz, 2H, H_{Ar}), 4.36 (t, J = 6.8 Hz, 4H, -COOCH_2-), 1.80 (m, 4H, -COOCH$_2$-CH_2), 1.47 (m, 4H, -CH_2-CH$_3$), 1.00 (t, J = 7.37, 6H, -CH$_2$-CH_3). ^{13}C NMR was not obtained due to poor solubility. MS (MALDI-TOF) m/z = 523.99 $[M+H]^+$, calcd. for $C_{32}H_{27}O_7$ = 523.18.

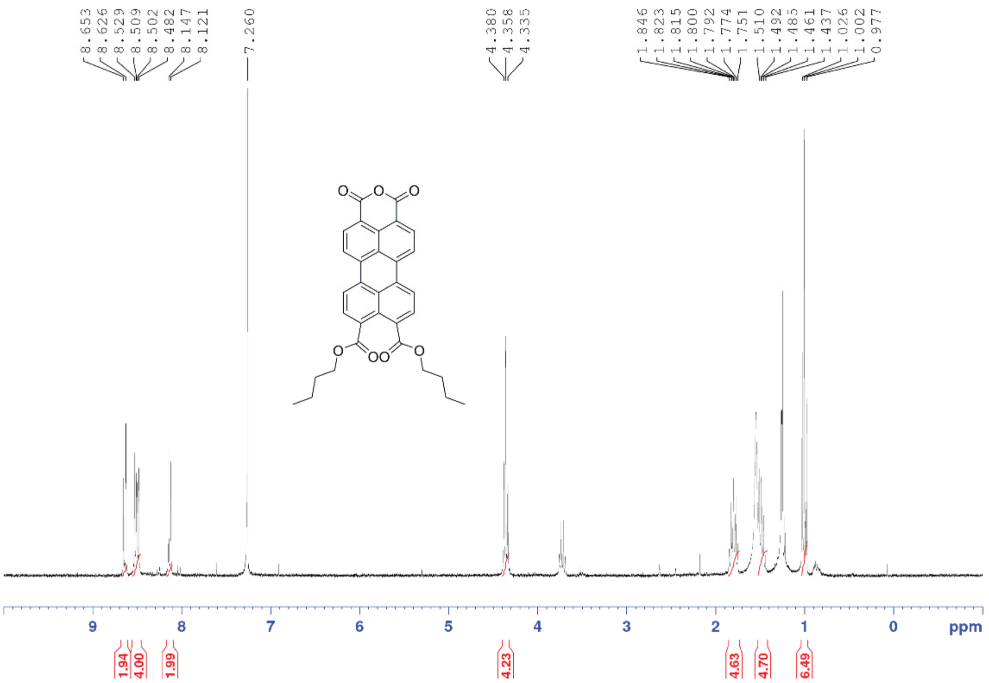

Supplementary Figure 24. ^{1}H NMR spectrum of PDMA in $CDCl_3$.

Synthesis of N-*(4-butanoic acid)-perylene-3,4,9,10-tetracarboxylic dibutyl ester monoimide (PDMI-acid)*

Perylene-3,4,9,10-tetracarboxylic dibutyl ester monoanhydride (PDMA, 1.55 g, 2.97 mmol), γ-aminobutyric acid (458 mg, 4.44 mmol) and triethylamine (1.03 g, 5.96 mmol) were dissolved in 5 mL of *N*,*N*-dimethylformamide and stirred at 80 °C. Progress of the reaction was monitored by TLC (dichloromethane:methanol, 9.5:0.5, v/v). After 3 h, the crude reaction mixture was allowed to cool to room temperature. 2 M aqueous hydrochloric acid was added dropwise to the cooled mixture and the resulting dark red precipitate formed was collected by centrifugation. The precipitate was washed with copious amounts of 2 M aqueous hydrochloric acid and water, air-dried at room temperature overnight and further dried under high vacuum before being purified by silica column chromatography (gradient, dichloromethane:acetone:formic acid, 9:1:0.1, v/v/v to dichloromethane:methanol:formic acid 9.5:0.5:0.1, v/v/v) to yield N-*(4-butanoic acid)-perylene-3,4,9,10-tetracarboxylic dibutyl ester monoimide* (PDMI-acid) as a very dark red solid (1.28 g, 71%). ^{1}H NMR (600 MHz, $(CD_3)_2SO$)) δ (ppm): 8.37 (d, *J* = 8.0 Hz, 2H, H_{Ar}), 8.23 (m, 4H, H_{Ar}), 7.88(d, *J* = 7.9 Hz, 2H, H_{Ar}), 4.27 (t, *J* = 6.7 Hz, 4H, -COOCH_2-), 4.02 (t, *J* = 6.9 Hz, 2H, -CH_2-COOH), 2.33 (t, *J* = 7.3, 2H, -NCH_2-), 1.91 (m, 2H, NCH_2-CH_2-), 1.75 (m, 4H, -COOCH_2-CH_2-), 1.47 (m, 4H, -CH_2-CH_3), 0.98 (t, *J* = 7.4, 6H, -CH_2-CH_3). ^{13}C NMR (151 MHz, $(CD_3)_2SO$) δ (ppm): 174.1, 167.4, 162.7, 134.2, 131.4, 131.0 130.6, 130.1, 128.0, 128.0, 124.8, 123.2, 122.4, 121.4, 64.9, 40.1, 31.5, 30.1, 23.0, 18.8, 13.7. MS (MALDI-TOF) *m/z* = 609.22 $[M+H]^+$, calcd. for $C_{36}H_{34}NO_8$ = 608.23.

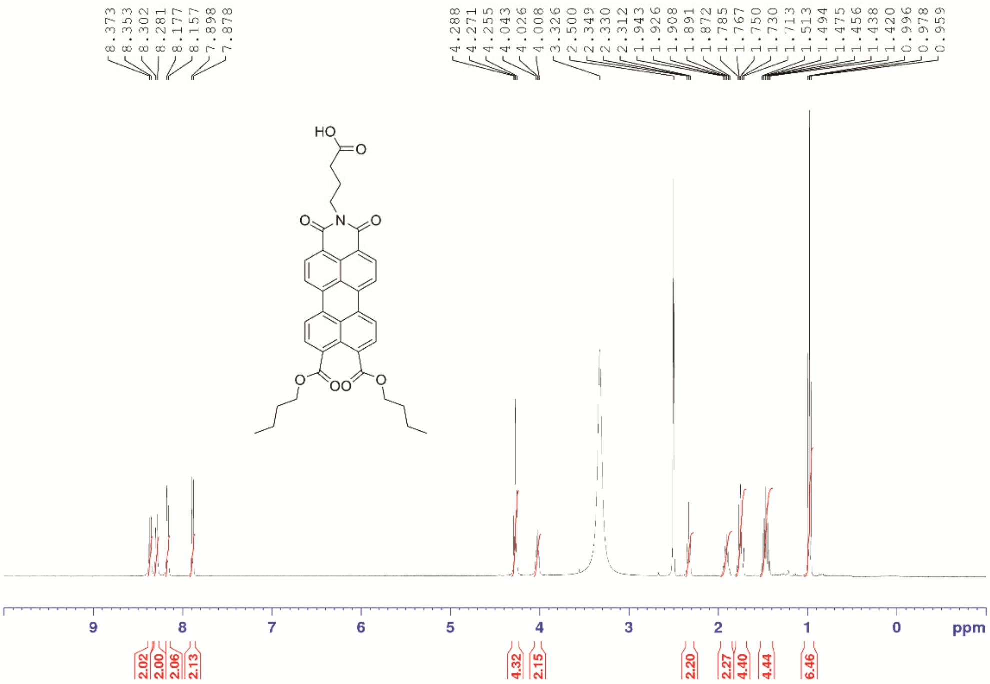

Supplementary Figure 25. ^{1}H NMR spectrum of PDMI-acid in $(CD_3)_2SO$.

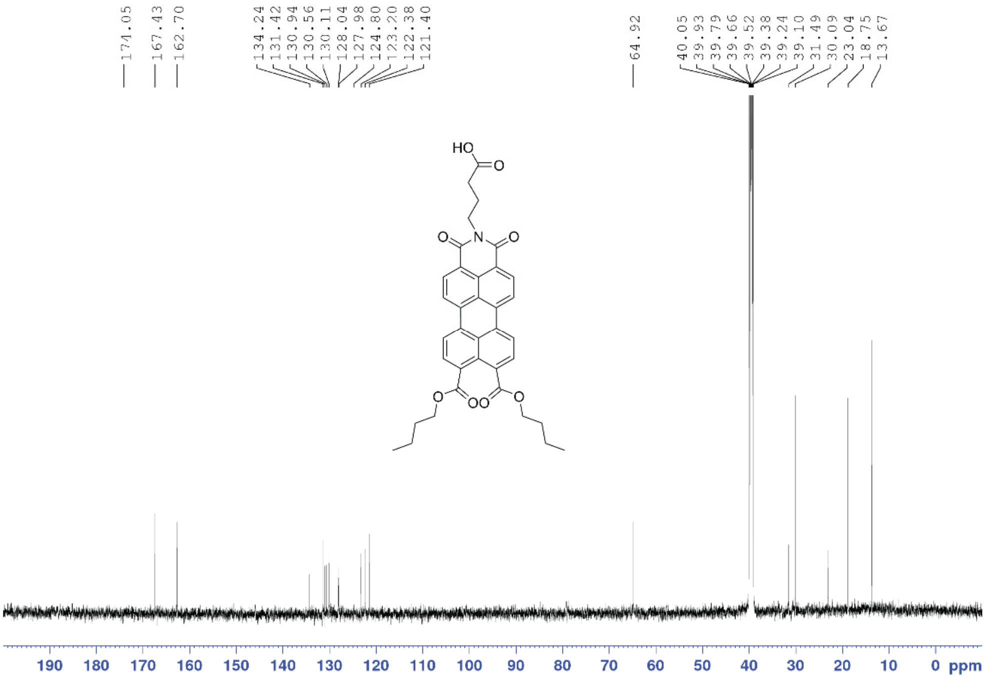

Supplementary Figure 26. ^{13}C NMR spectrum of PDMI-acid in $(CD_3)_2SO$.

Synthesis of N*-(pentafluorophenyl butyl ester)-perylene-3,4,9,10-tetracarboxylic monoimide dibutyl ester (PDMI-PFP)*

N-(4-Butanoic acid)-perylene-3,4,9,10-tetracarboxylic monoimide dibutyl ester (PDMI-acid, 1.28 g, 2.11 mmol), pentafluorophenol (775 mg, 4.21 mmol) and 1-(3-dimethylaminopropyl)-3-ethylcarbodiimide hydrochloride (EDC●HCl) (606 mg, 3.16 mmol) were dissolved in 10 mL of dichloromethane and stirred under nitrogen for 25 h. The crude reaction mixture was diluted with 100 mL of dichloromethane and washed with 2 M aqueous hydrochloric acid (x3), saturated sodium bicarbonate solution (x3), water (x3) and brine (x2) before being dried over anhydrous magnesium sulphate. The organic phase was then concentrated *in vacuo* to yield N-*(pentafluorophenyl butyl ester)-perylene-3,4,9,10-tetracarboxylic monoimide dibutyl ester* (PDMI-PFP) as a dark red solid (1.43 g, 88%). ^{1}H NMR (300 MHz, $CDCl_3$) δ (ppm): 8.62 (d, *J* = 8.1, 2H, H_{Ar}), 8.45 (m, *4*H, H_{Ar}), 8.10 (d, *J* = 8.0, 2H, H_{Ar}), 4.36 (t, *J* = 6.8, 6H, -COOCH_2-), 2.86 (t, *J* = 7.5, 2H, -NCH_2-), 2.27 (m, 2H, -NCH$_2$-CH_2-), 1.80 (m, 4H, -COOCH$_2$-CH_2-), 1.49 (m, 4H, -CH_2-CH$_3$), 1.00 (t, *J* = 7.4, 6H, -CH$_2$-CH_3). ^{13}C NMR (75 MHz, $CDCl_3$) δ (ppm): 169.0, 168.4, 163.7, 135.7, 132.2, 132.0, 131.6, 130.4, 129.3, 129.1, 126.0, 122.8, 121.9, 121.9, 65.8, 39.4, 31.2, 30.8, 23.4, 19.4, 14.0 ^{19}F NMR (75 MHz, $CDCl_3$) δ (ppm): -152.47 (d, 2F), -158.17 (t, 1F), -162.42 (m, 2F). MS (MALDI-TOF) *m/z* = 774.47 $[M+H]^+$, calcd. for $C_{42}H_{33}F_5NO_8$ = 774.21.

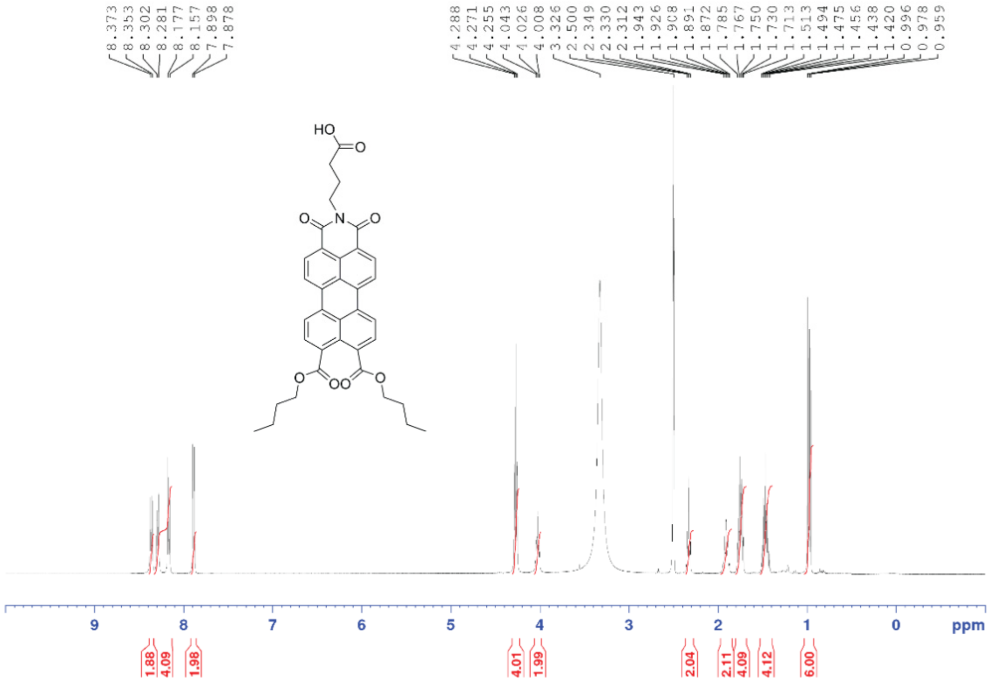

Supplementary Figure 27. ^{1}H NMR spectrum of PDMI-PFP in $CDCl_3$.

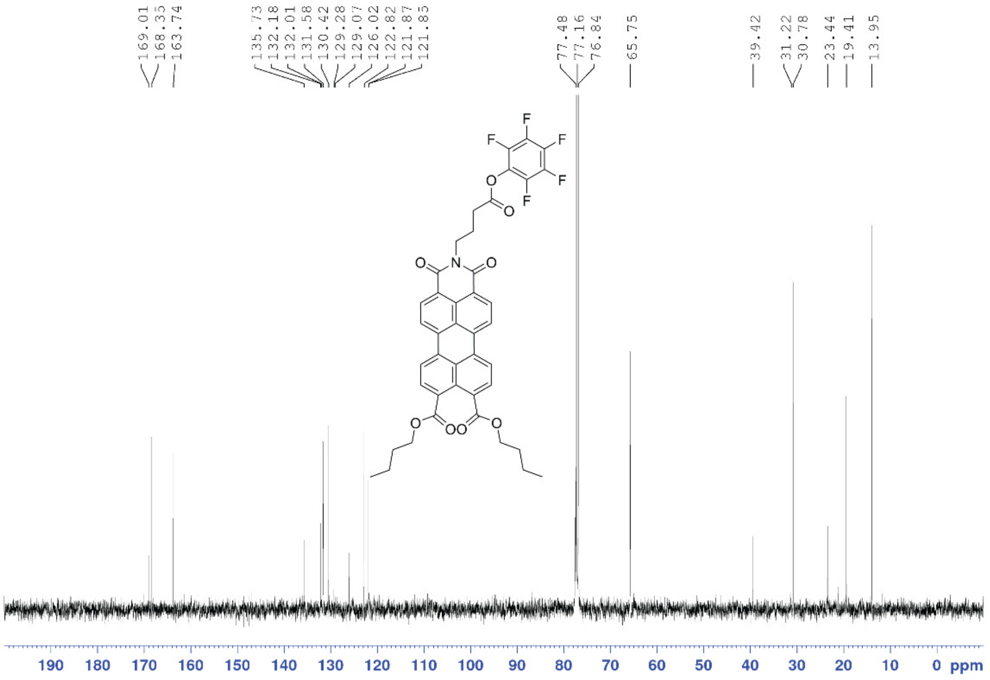

Supplementary Figure 28. ^{13}C NMR spectrum of PDMI-PFP in $CDCl_3$.

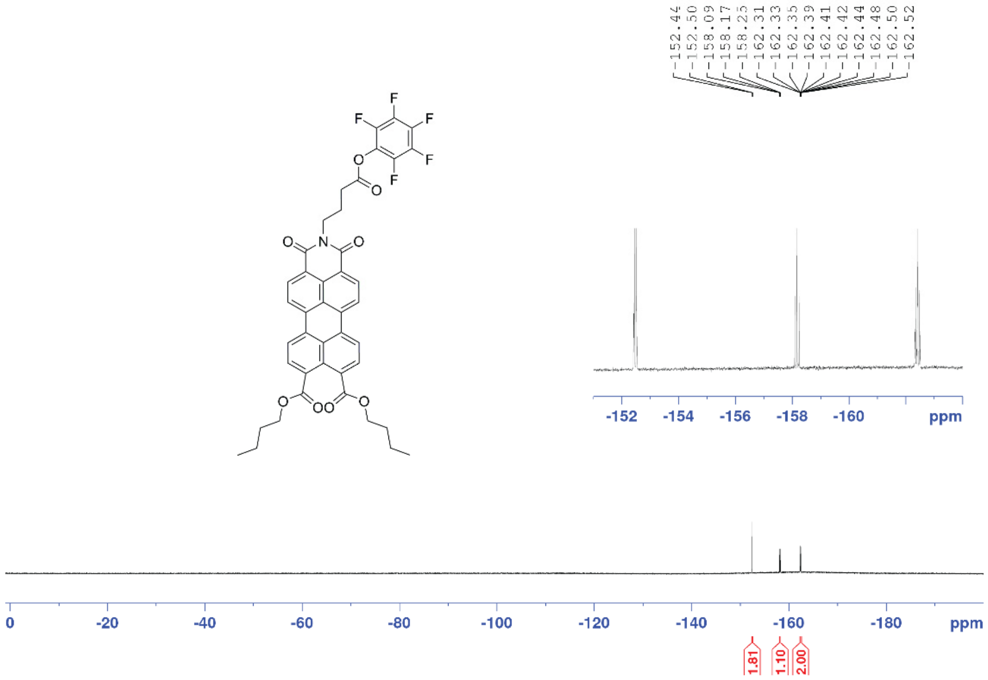

Supplementary Figure 29. ^{19}F NMR spectrum of PDMI-PFP in $CDCl_3$.

Synthesis and characterization of PEG_{43}-DDMAT and *t*-BocAEA

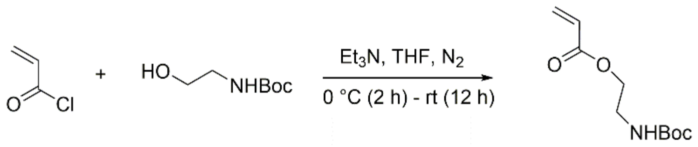

Supplementary Figure 30. Synthetic route to obtain the *t*-Boc-protected monomer *t*-BocAEA.

Synthesis of N-*(*tert*-butoxycarbonyl)aminoethyl acrylate (t-BocAEA)*

N-(*tert*-butoxycarbonyl)ethanolamine (6.00 g, 37.2 mmol) and triethylamine (3.77 g, 37.3 mmol) were dissolved in 13 mL of tetrahydrofuran and stirred under nitrogen at 0 °C (ice bath) for 20 min. To the solution was then added acryloyl chloride (3.34 g, 36.9 mmol) dropwise over 1 h. Following the addition, the mixture was stirred at 0 °C for 2 h before being allowed to warm to room temperature, and stirred for a further 12 h, during which the formation of an off-white precipitate was observed. The precipitate was collected by suction filtration and purified by silica column chromatography (dichloromethane:ethyl acetate, 7:3, v/v) to yield an off-white oil that was further purified by recrystallization from hexane to yield N-*(*tert-*butoxycarbonyl)aminoethyl acrylate* (*t*-BocAEA) as a white solid (5.33 g, 67%). ^{1}H NMR (300 MHz, $CDCl_3$) δ (ppm): 6.43 (dd, *J* =17.3 Hz, 2H, C*H*$_2$=CH-), 6.13 (dd, *J* = 17.3, 2H CH_2=C*H*-), 5.85 (dd, *J* = 10.4, 1H, C*H*$_2$=CH-), 4.80 (br s, 1H, -N*H*), 4.22 (t, 2H, *J* = 5.3, -COOC*H*$_2$-), 3.43 (q, 2H, -COOCH$_2$-C*H*$_2$), 1.44 (s, 9H, C(C*H*$_3$)$_3$). ^{13}C NMR (75 MHz, $CDCl_3$) δ (ppm): 166.2, 155.9, 131.4, 128.2, 79.8, 64.0, 39.8, 28.5. MS (ESI) *m/z* = 238.00 $[M+Na]^+$, calcd. for $C_{10}H_{17}NO_4Na$ = 238.10. These results are in good agreement with those reported in literature.[3]

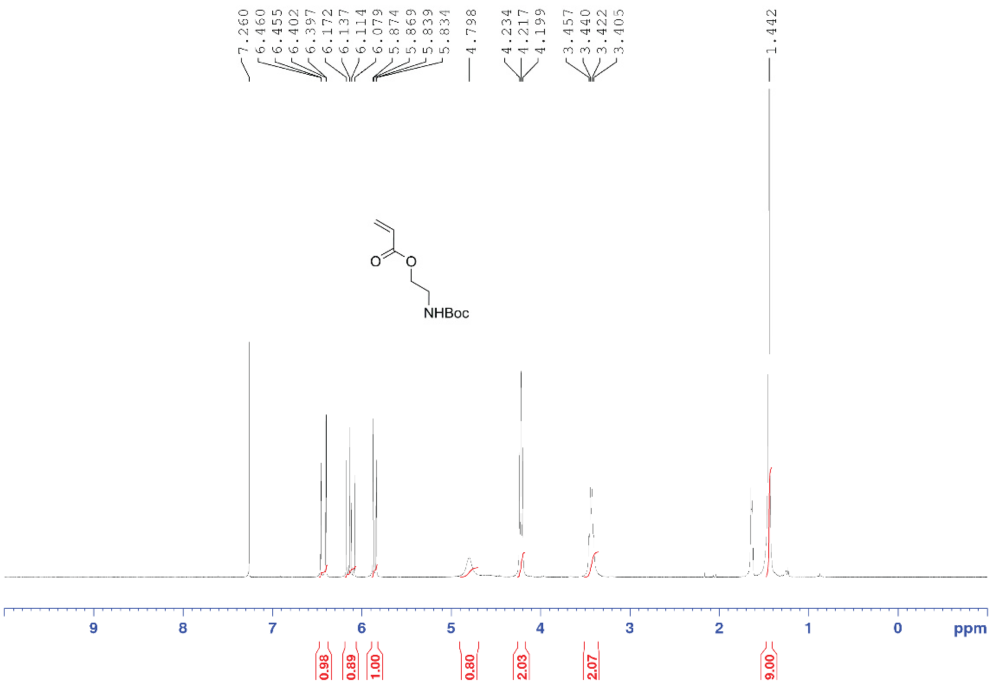

Supplementary Figure 31. ^{1}H NMR spectrum of *t*-BocAEA in $CDCl_3$.

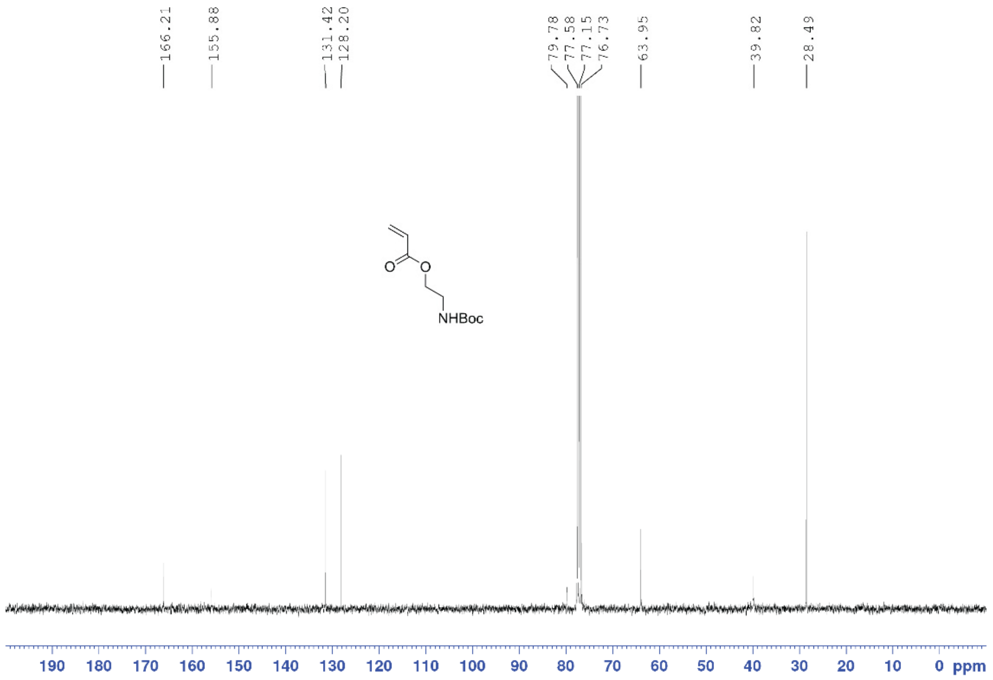

Supplementary Figure 32. ^{13}C NMR spectrum of *t*-BocAEA in $CDCl_3$.

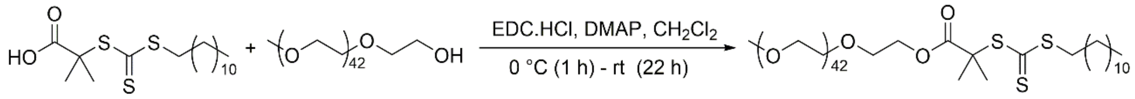

Supplementary Figure 33. Synthetic route to obtain the PEG_{43}-based macro-RAFT agent (PEG_{43}-DDMAT).

Synthesis of poly(ethylene glycol) methyl ether 2-(dodecylthiocarbonothioylthio)-2-methylpropionate (PEG$_{43}$*-DDMAT)*

2-(Dodecylthiocarbonothioylthio)-2-methylpropionic acid (DDMAT) (1.02 g, 2.80 mmol) and 1-(3-dimethylaminopropyl)-3-ethylcarbodiimide hydrochloride (EDC●HCl) (586 mg, 3.04 mmol) were dissolved in 10 mL of dichloromethane and stirred under nitrogen in an ice bath (0 °C) for 20 min. To the yellow solution was then added another solution containing poly(ethylene glycol)$_{43}$ (PEG_{43}-OH, 4.80 g, 2.53 mmol) and 4-dimethylaminopyridine (DMAP) (123 mg, 1.01 mmol) in 9 mL of dichloromethane. The mixture was stirred at 0 °C for 1 h before being allowed to warm to room temperature, and stirred for a further 22 h. The crude reaction mixture was diluted with 50 mL of dichloromethane and washed with 2 M aqueous hydrochloric acid (x3), water (x3) and brine (x3) before being dried over anhydrous magnesium sulphate. The organic phase was concentrated *in vacuo* to yield a yellow oil that was redissolved in ~15 mL of tetrahydrofuran and precipitated into cold hexane. The resulting precipitate was collected by centrifugation, redissolved in ~15 mL of tetrahydrofuran and precipitated into cold hexane again. The purification process was repeated three times in total. The purified product was collected and dried under high vacuum to yield *poly(ethylene glycol) methyl ether 2-(dodecylthiocarbonothioylthio)-2-methylpropionate* (PEG_{43}-DDMAT) as a light yellow solid (4.25 g, 69 %). $M_{n,GPC}$ = 3,500 g mol^{-1}; $Đ$ = 1.04.

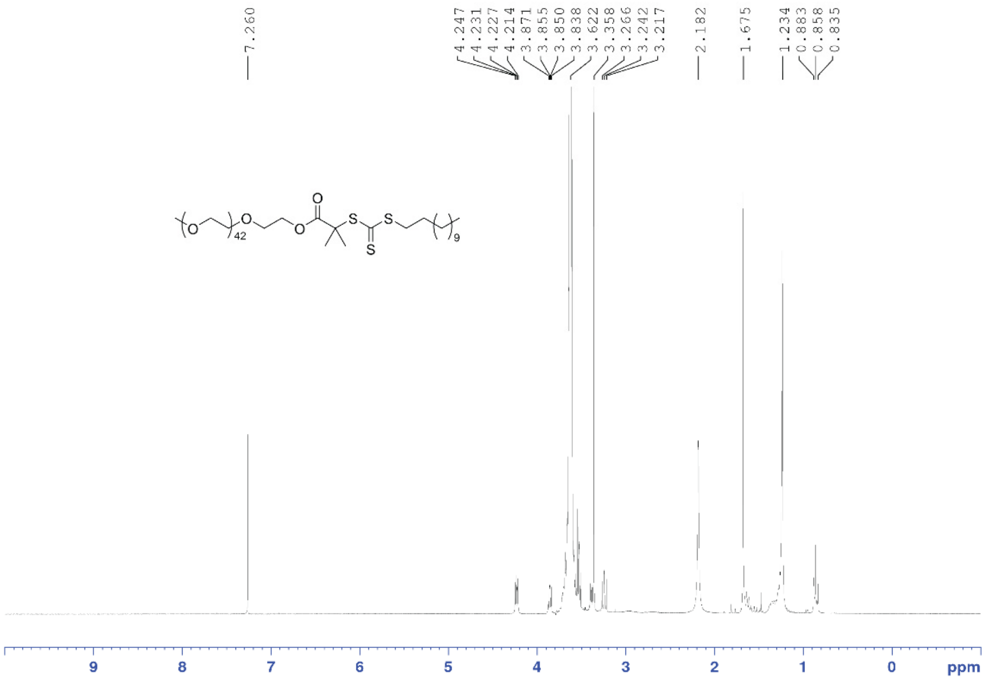

Supplementary Figure 34. ^{1}H NMR spectrum of PEG_{43}-DDMAT in $CDCl_3$.

Synthesis and characterization of PEG$_{43}$-*b*-P(NIPAM$_{21}$-*co*-AEA$_9$)

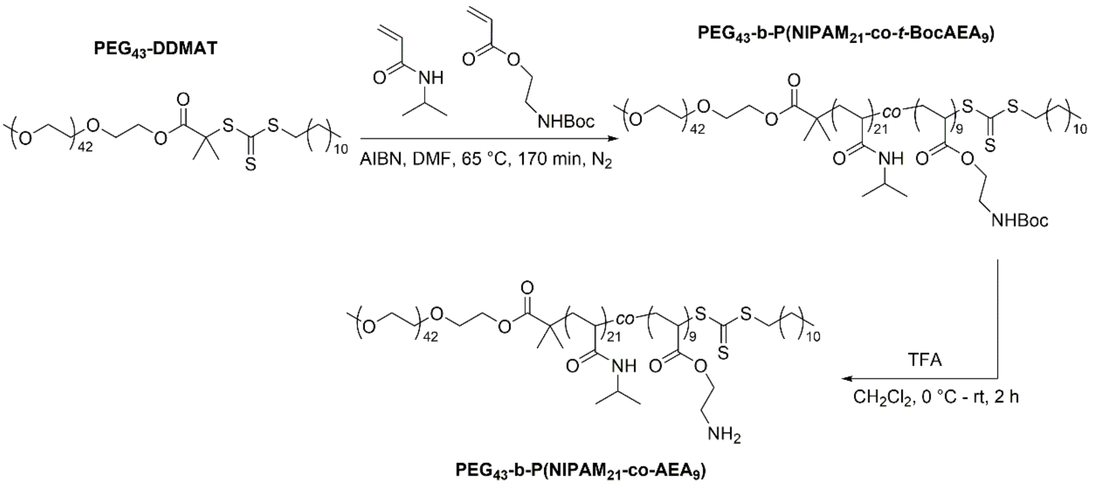

Supplementary Figure 35. Synthetic route to obtain the amine-containing diblock terpolymer PEG$_{43}$-*b*-P(NIPAM$_{21}$-*co*-AEA$_9$).

Synthesis of PEG$_{43}$-b-P(NIPAM$_{21}$-co-t-BocAEA$_9$) by RAFT (co)polymerization

N-isopropylacrylamide (NIPAM) (395 mg, 3.46 mmol), *t*-BocAEA (300 mg, 1.39 mmol), poly(ethylene glycol) methyl ether 2-(dodecylthiocarbonothioylthio)-2-methylpropionate (PEG$_{43}$-DDMAT, 0.32 g, 0.14 mmol) and 2,2'-azobis(2-methylpropionitrile) (AIBN, 0.2 M in toluene) (4.58 mg, 27.9 µmol) were dissolved in 900 µL of *N*,*N*-dimethylformamide and degassed by purging with nitrogen for 25 min. The degassed mixture was then placed in a pre-heated oil bath at 65 °C. After 170 min, the reaction vessel was exposed to air and quenched in an ice bath. The crude reaction mixture was diluted in minimal amounts of tetrahydrofuran and precipitated into cold hexane:diethyl ether (2:1, v/v). The resulting precipitate was collected by centrifugation, redissolved in minimal amounts of tetrahydrofuran and precipitated into cold hexane:diethyl ether (2:1, v/v) again. The purification process was repeated four times in total. The purified product was collected and dried under high vacuum to yield *PEG$_{43}$-b-P(NIPAM$_{21}$-co-t-BocAEA$_9$)* as a light yellow solid (695 mg). The number of NIPAM and *t*-BocAEA

repeating units in the diblock terpolymer and the number-average molecular weight by NMR ($M_{n,NMR}$) were not determined in this step due to overlapping peaks in the ^{1}H NMR spectra as shown in Supplementary Fig. 36. $M_{n,GPC}$ = 14,000 g mol^{-1}; $Đ$ = 1.07.

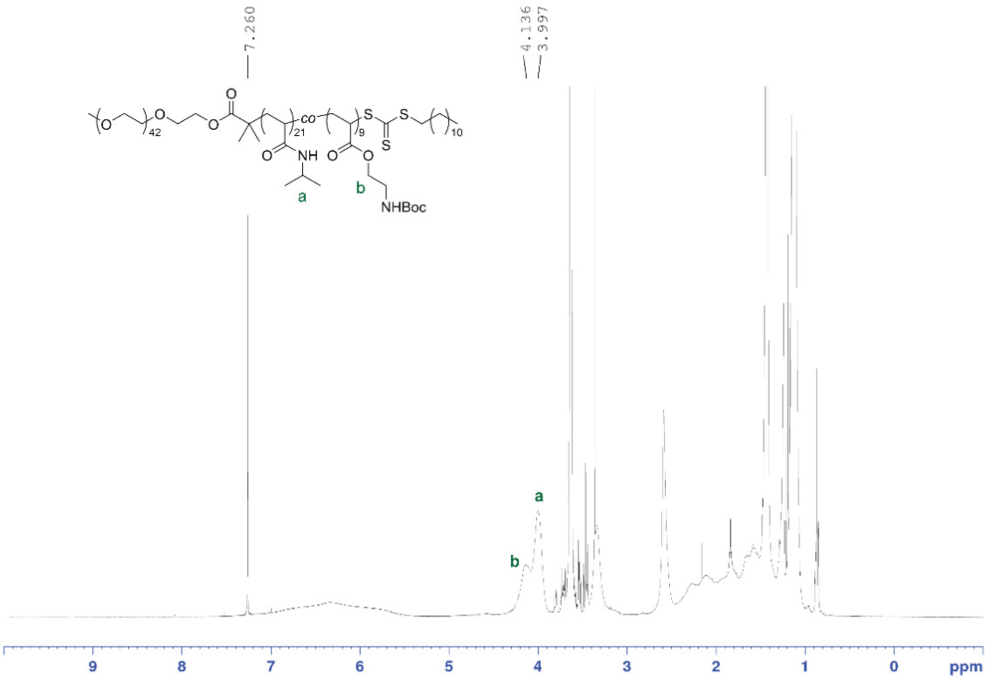

Supplementary Figure 36. ^{1}H NMR spectrum of PEG$_{43}$-*b*-P(NIPAM$_{21}$-*co*-*t*-BocAEA$_9$) in $CDCl_3$.

Deprotection of PEG$_{43}$-b-P(NIPAM$_{21}$-co-t-BocAEA$_9$)

A solution of PEG$_{43}$-*b*-P(NIPAM$_{21}$-*co*-*t*-BocAEA$_9$) (100 mg) in 3 mL of dichloromethane was stirred at 0 °C (ice bath) for 10 min. 1 mL of trifluoroacetic acid was added dropwise to the solution over 2 min. Following the addition, the mixture was further stirred at 0 °C. After 2 h, the crude reaction mixture was concentrated *in vacuo* (repeated three times using dichloromethane to azeotropically remove trifluoroacetic acid). The resulting yellow oil was

then dissolved in minimal amounts of tetrahydrofuran and precipitated into hexane. The resulting precipitate was collected by centrifugation and dried under high vacuum to yield *PEG43-b-P(NIPAM21-co-AEA9)* as a light yellow solid. The number of NIPAM and AEA repeating units in the deprotected diblock terpolymer were determined by comparing the peak integrals in the ^{1}H NMR spectra as shown in Supplementary Fig. 37. $M_{n,NMR}$ = 5,675 g mol^{-1}; $M_{n,GPC}$ = 4,700 g mol^{-1}; $Đ$ = N/A (GPC analysis gave an unreliable value of 1.01).

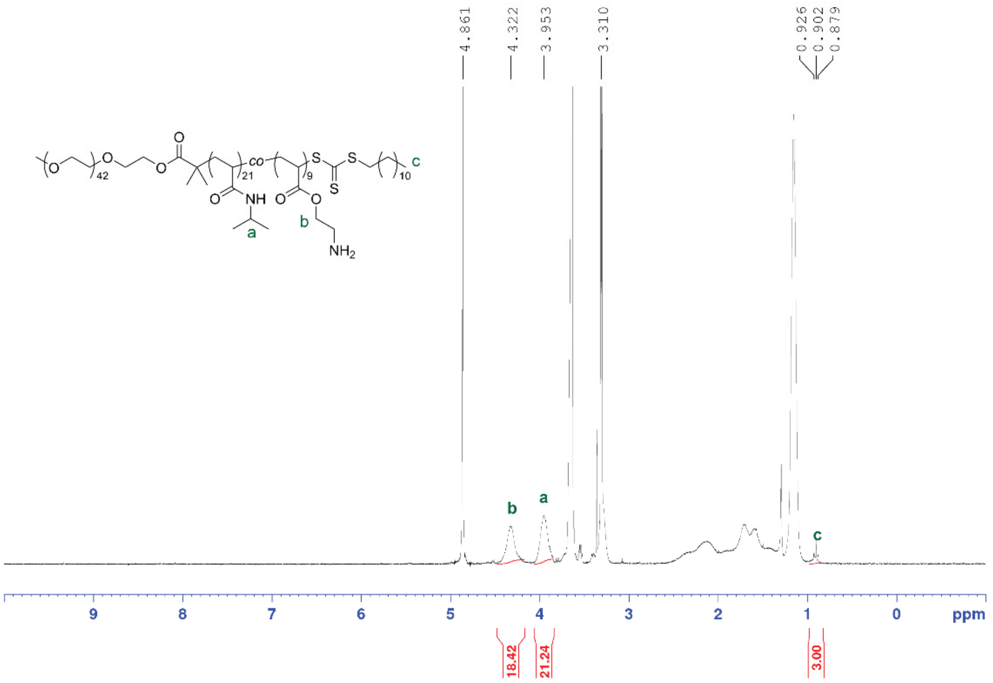

Supplementary Figure 37. ^{1}H NMR spectrum of PEG$_{43}$-*b*-P(NIPAM$_{21}$-*co*-AEA$_9$) in CD_3OD.

Synthesis and characterization of PEG$_{43}$-*b*-P(NIPAM$_{22}$-*co*-PDMI$_5$)

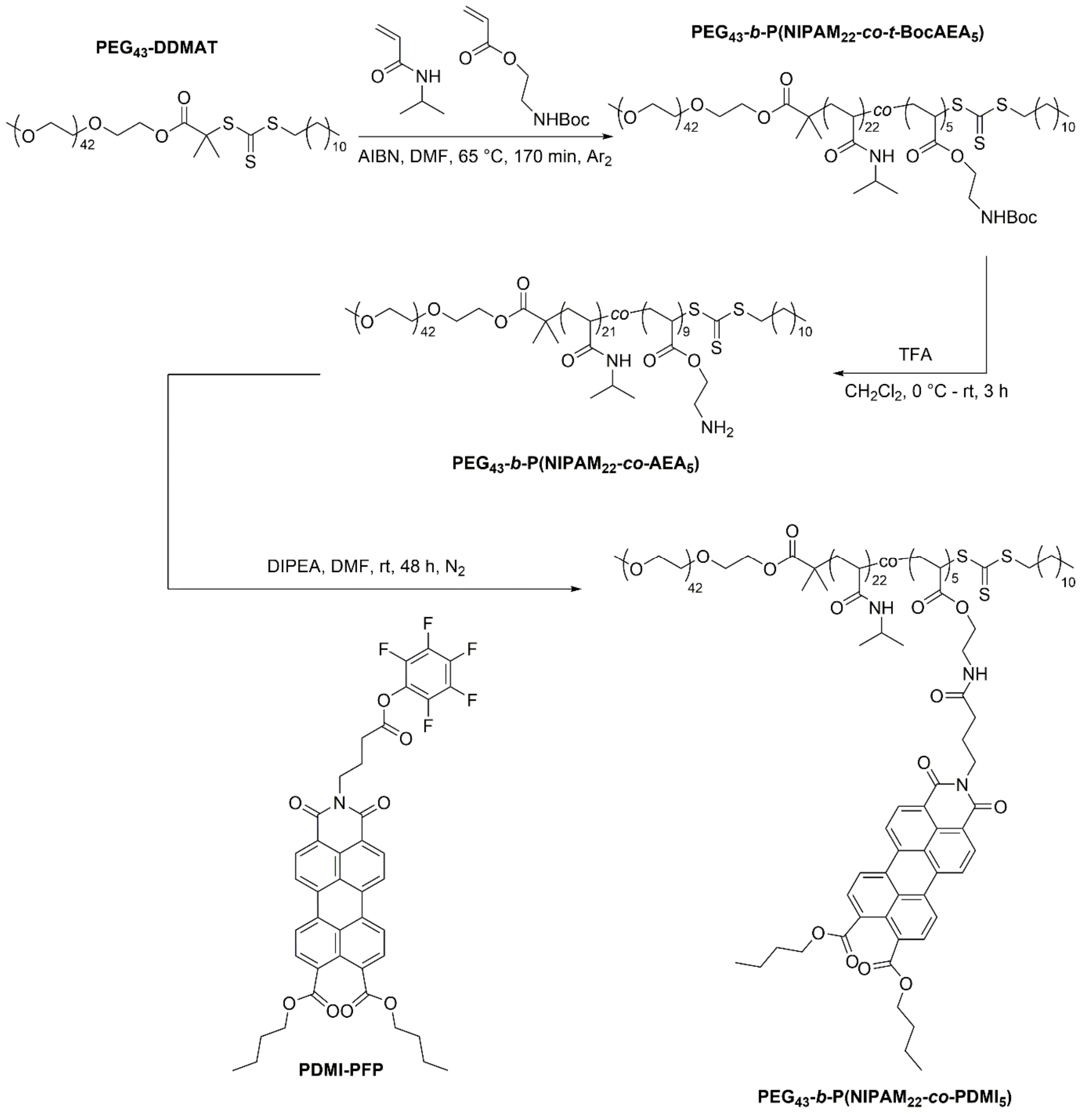

Supplementary Figure 38. Synthetic route to obtain the diblock terpolymer PEG$_{43}$-*b*-P(NIPAM$_{22}$-*co*-PDMI$_5$).

Synthesis of PEG$_{43}$-b-P(NIPAM$_{22}$-co-t-BocAEA$_5$) by RAFT (co)polymerization

N-isopropylacrylamide (NIPAM) (394 mg, 3.48 mmol), *t*-BocAEA (150 mg, 6.97 mmol), poly(ethylene glycol) methyl ether 2-(dodecylthiocarbonothioylthio)-2-methylpropionate

(PEG_{43}-DDMAT, 0.32 g, 0.14 mmol) and 2,2'-azobis(2-methylpropionitrile) (AIBN, 0.2 M in toluene) (4.57 mg, 27.9 µmol) were dissolved in 900 µL of *N*,*N*-dimethylformamide and degassed by purging with argon for 20 min. The degassed mixture was then placed in a pre-heated oil bath at 65 °C. After 170 min, the reaction vessel was exposed to air and quenched in an ice bath. The crude reaction mixture was diluted in minimal amounts of tetrahydrofuran and precipitated into cold hexane:diethyl ether (2:1, v/v). The resulting precipitate was collected by centrifugation, redissolved in minimal amounts of tetrahydrofuran and precipitated into cold hexane:diethyl ether (2:1, v/v) again. The purification process was repeated four times in total. The purified product was collected and dried under high vacuum to yield PEG_{43}-*b*-P($NIPAM_{22}$-*co*-*t*-$BocAEA_{5}$) as a light yellow solid (555 mg). The number of NIPAM and *t*-BocAEA repeating units in the diblock terpolymer and the number-average molecular weight by NMR ($M_{n,NMR}$) were not determined in this step due to overlapping peaks in the ^{1}H NMR spectrum as shown in Supplementary Fig. 39. $M_{n,GPC}$ = 11,000 g mol^{-1}; $Đ$ = 1.04.

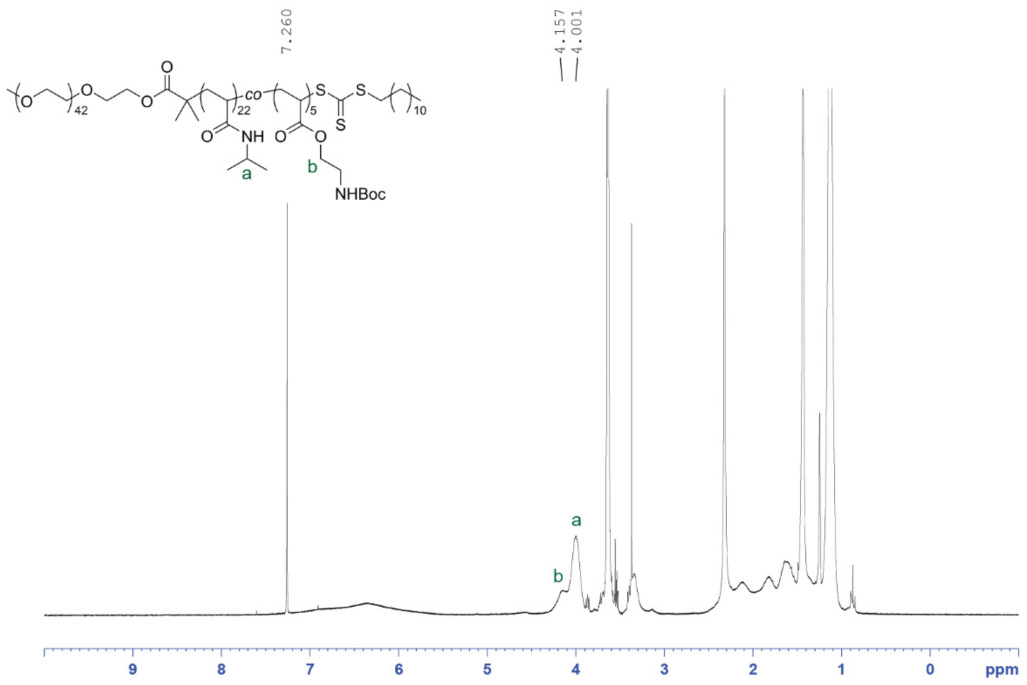

Supplementary Figure 39. ^{1}H NMR spectrum of PEG$_{43}$-*b*-P(NIPAM$_{22}$-*co*-*t*-BocAEA$_5$) in $CDCl_3$.

Deprotection of PEG$_{43}$-b-P(NIPAM$_{22}$-co-t-BocAEA$_5$)

A solution of *PEG$_{43}$-b-P(NIPAM$_{22}$-co-t-BocAEA$_5$)* (50.3 mg) in 4 mL of dichloromethane was stirred at 0 °C (ice bath) for 10 min. 2 mL of trifluoroacetic acid was added dropwise to the solution over 2 min. Following the addition, the mixture was further stirred at 0 °C. After 3 h, the crude reaction mixture was concentrated *in vacuo* (repeated three times using dichloromethane to azeotropically remove trifluoroacetic acid). The resulting yellow oil was then dissolved in minimal amounts of tetrahydrofuran and precipitated into hexane. The resulting precipitate was collected by centrifugation and dried under high vacuum to yield *PEG$_{43}$-b-P(NIPAM$_{22}$-co-AEA$_5$)* as a light yellow solid. The number of NIPAM and AEA repeating units in the deprotected diblock terpolymer were determined by comparing the peak

integrals in the ^{1}H NMR spectra as shown in Supplementary Fig. 40. $M_{n,NMR}$ = 5,334 g mol^{-1}; $M_{n,GPC}$ = N/A; $Đ$ = N/A (GPC analysis gave unreliable values).

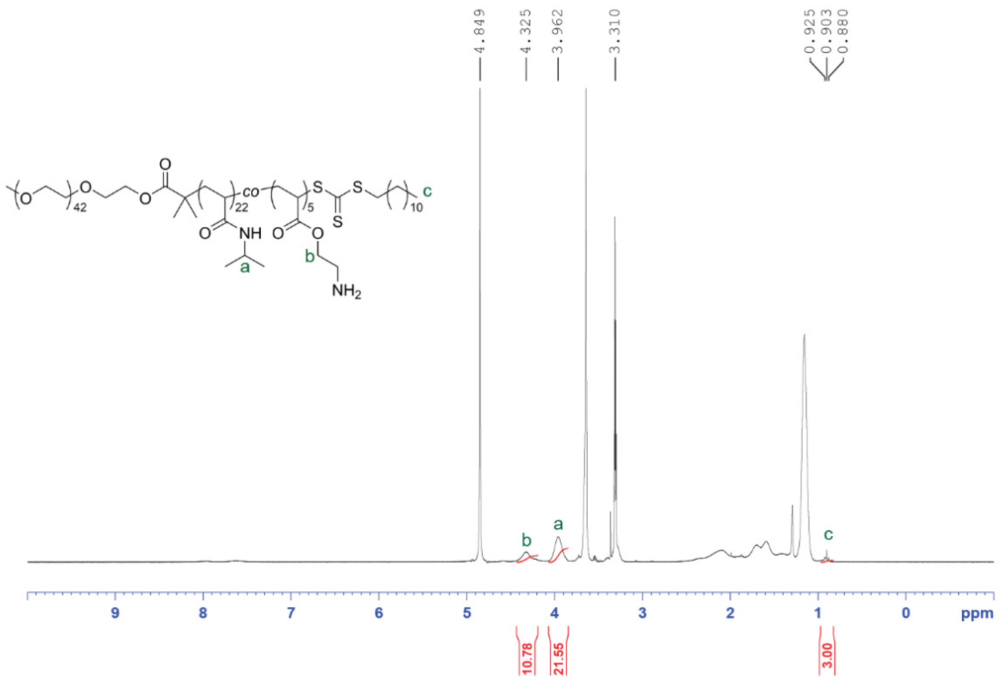

Supplementary Figure 40. ^{1}H NMR spectrum of PEG$_{43}$-*b*-P(NIPAM$_{22}$-*co*-AEA$_5$) in MeOD.

Synthesis of PEG$_{43}$-b-P(NIPAM$_{22}$-co-PDMI$_5$)

The *t*-Boc deprotected diblock terpolymer PEG$_{43}$-*b*-P(NIPAM$_{21}$-*co*-AEA$_9$) (46.0 mg, 8.63 µmol), *N*-(pentafluorophenyl butyl ester)-perylene-3,4,9,10-tetracarboxylic monoimide dibutyl ester (PDMI-PFP, 40 mg, 0.051 mmol) and *N*,*N*-diisopropylethylamine (7 mg, 0.054 mmol) were dissolved in 5 mL of *N*,*N*-dimethylformamide and stirred under nitrogen. Progress of the reaction was monitored by TLC (dichloromethane:acetone, 9.5:0.5, v/v). After 48 h, the crude reaction mixture was purified by size-exclusion chromatography (Bio-Beads S-X1, THF). The initial red elution band corresponding to a higher molecular weight product was

collected and further purified by dialysis (3.5 kDa MWCO) against THF:ethanol (1:1, v/v) followed by dichloromethane:ethanol (1:1, v/v). Further purification by dialysis was necessary due to the high tendency of unreacted PDMI-PFP to aggregate with the desired product, causing it to co-elute in the initial red elution band. The dialyzed product was then concentrated *in vacuo* and further dried under high vacuum to yield *PEG$_{43}$-b-P(NIPAM$_{22}$-co-PDMI$_5$)* as a dark red solid (52 mg, 74%). $M_{\text{n,NMR}}$ = 8,278 g mol^{-1}; $M_{n,\text{GPC}}$ = 12,900 g mol^{-1}; $Đ$ = 1.05.

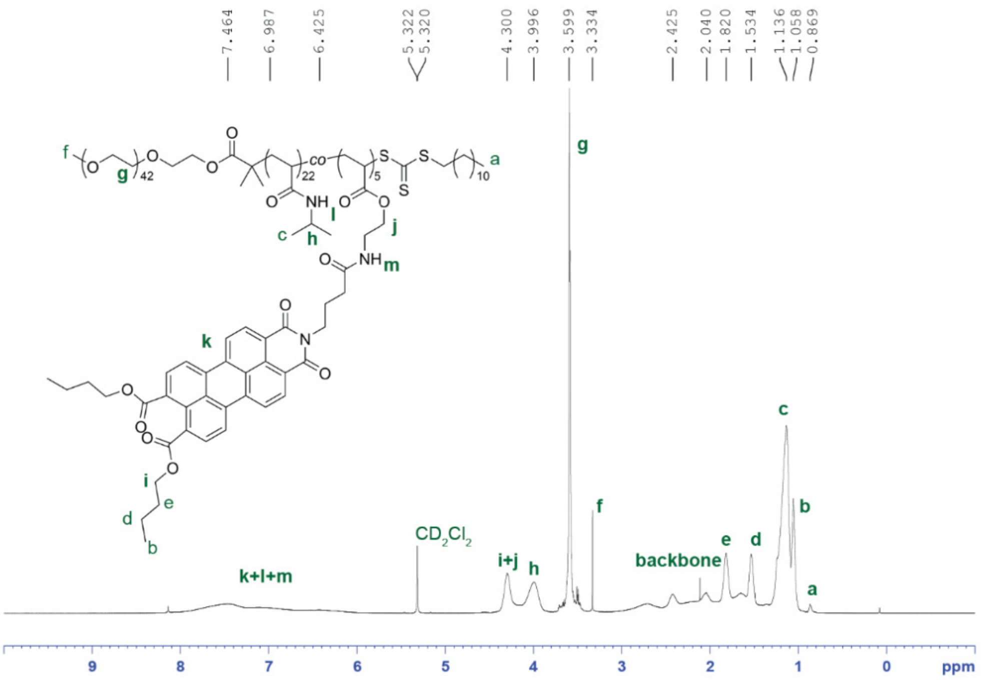

Supplementary Figure 41. ^{1}H NMR spectrum of PEG$_{43}$-*b*-P(NIPAM$_{22}$-*co*-PDMI$_5$) in CD_2Cl_2.

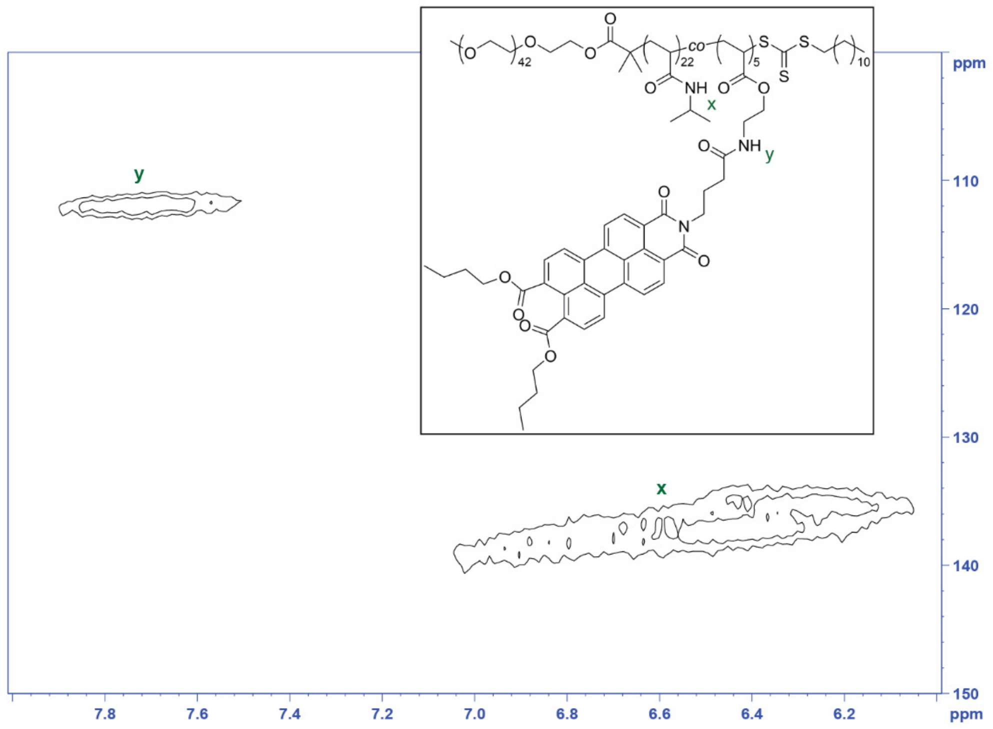

Supplementary Figure 42. ^1H-^{15}N HSQC NMR spectrum of PEG$_{43}$-*b*-P(NIPAM$_{22}$-*co*-PDMI$_5$) in CD_2Cl_2 showing cross-peaks corresponding to the two different amide protons of the diblock terpolymer.

Supplementary Table 3. Integration values and relative integration values for ^1H-^{15}N cross-peaks **x** and **y** shown in Supplementary Fig. 42. The degree of dye functionalization on PEG$_{43}$-*b*-P(NIPAM$_{22}$-*co*-PDMI$_5$) was obtained by multiplying the known number of repeating units of NIPAM (i.e. 22 repeat units, determined using integrals from the ^1H NMR spectrum (Supplementary Fig. 40) of PEG-*b*-P(NIPAM$_{22}$-*co*-AEA$_5$)) by the relative integration value of 0.22 (*i.e.* $22 \times 0.22 = 4.84 \approx 5$ repeat units of PDMI).

Peak	Integral (abs)	Integral (rel)	ν(F2) (ppm)	ν(F1) [ppm]
**y**	9.51E+06	0.22	7.66	111.9
**x**	4.33E+07	1.00	6.61	136.9

Synthesis and characterization of PEG_{43}-*b*-P($NIPAM_{23}$-*co*-$PDMI_{19}$)

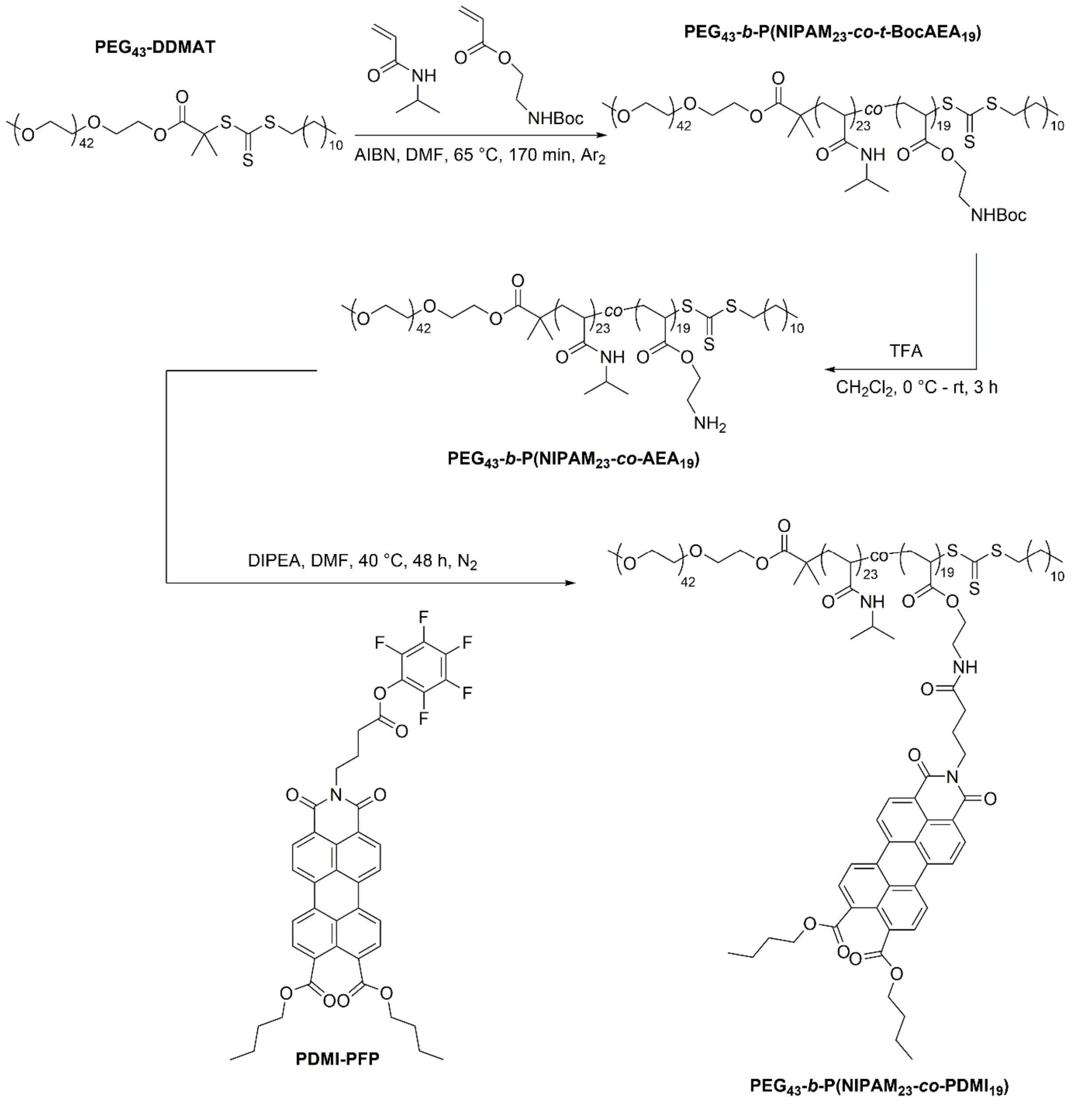

Supplementary Figure 43. Synthetic route to obtain the diblock terpolymer PEG_{43}-*b*-P($NIPAM_{23}$-*co*-$PDMI_{19}$).

Synthesis of PEG_{43}-b-P($NIPAM_{23}$-co-t-$BocAEA_{19}$) by RAFT (co)polymerization

N-isopropylacrylamide (NIPAM) (394 mg, 3.48 mmol), *t*-BocAEA (600 mg, 2.79 mmol), poly(ethylene glycol) methyl ether 2-(dodecylthiocarbonothioylthio)-2-methylpropionate (PEG_{43}-DDMAT, 0.32 g, 0.14 mmol) and 2,2'-azobis(2-meSthylpropionitrile) (AIBN, 0.2 M in toluene) (4.57 mg, 27.9 µmol) were dissolved in 900 µL of *N*,*N*-dimethylformamide and degassed by purging with argon for 20 min. The degassed mixture was then placed in a pre-heated oil bath at 65 °C. After 170 min, the reaction vessel was exposed to air and quenched in an ice bath. The crude reaction mixture was diluted in minimal amounts of tetrahydrofuran and precipitated into cold hexane:diethyl ether (2:1, v/v). The resulting precipitate was collected by centrifugation, redissolved in minimal amounts of tetrahydrofuran and precipitated into cold hexane:diethyl ether (2:1, v/v) again. The purification process was repeated four times in total. The purified product was collected and dried under high vacuum to yield *PEG_{43}-b-P($NIPAM_{23}$-co-t-$BocAEA_{19}$)* as a light yellow solid (891 mg). The number of NIPAM and *t*-BocAEA repeating units in the diblock terpolymer and the number-average molecular weight by NMR ($M_{n,NMR}$) were not determined in this step due to overlapping peaks in the ^{1}H NMR spectra as shown in Supplementary Fig. 44. $M_{n,GPC}$ = 14,800 g mol^{-1}; $Đ$ = 1.07.

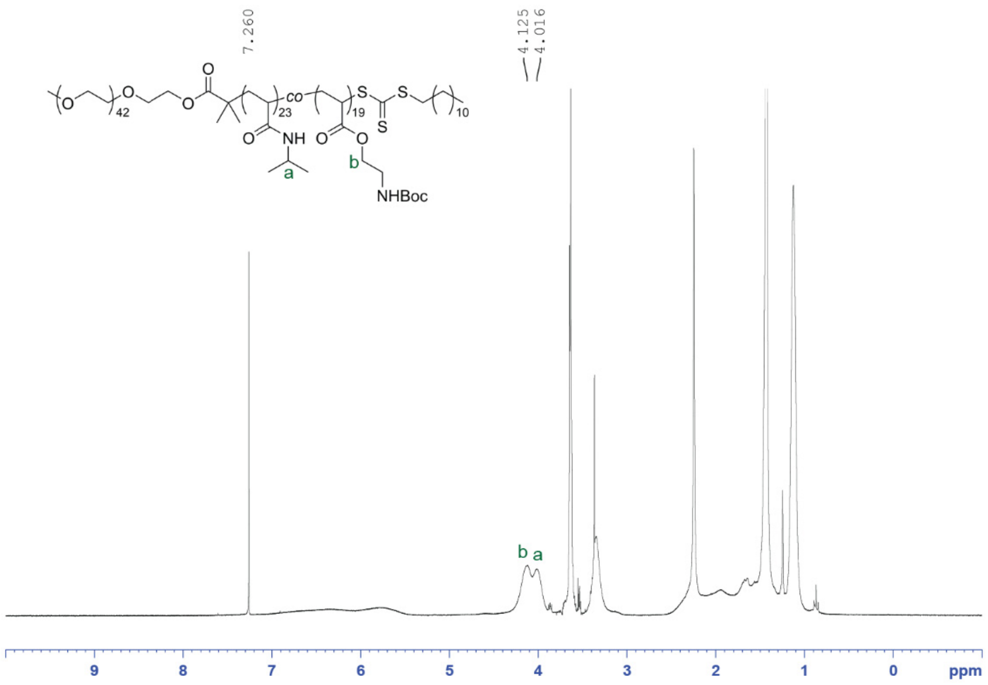

Supplementary Figure 44. ^{1}H NMR spectrum of PEG$_{43}$-*b*-P(NIPAM$_{23}$-*co*-*t*-BocAEA$_{19}$) in CDCl$_3$.

Deprotection of PEG$_{43}$-b-P(NIPAM$_{23}$-co-t-BocAEA$_{19}$)

A solution of *PEG$_{43}$-b-P(NIPAM$_{23}$-co-t-BocAEA$_{19}$)* (102 mg) in 5 mL of dichloromethane was stirred at 0 °C (ice bath) for 10 min. 2 mL of trifluoroacetic acid was added dropwise to the solution over 2 min. Following the addition, the mixture was further stirred at 0 °C. After 3 h, the crude reaction mixture was concentrated *in vacuo* (repeated three times using dichloromethane to azeotropically remove trifluoroacetic acid). The resulting yellow oil was then dissolved in minimal amounts of tetrahydrofuran and precipitated into hexane. The resulting precipitate was collected by centrifugation and dried under high vacuum to yield *PEG$_{43}$-b-P(NIPAM$_{23}$-co-AEA$_{19}$)* as a light yellow solid. The number of NIPAM and AEA repeating units in the deprotected diblock terpolymer were determined by comparing the peak

integrals in the ^{1}H NMR spectra as shown in Supplementary Fig. 45. $M_{n,NMR}$ = 7,068 g mol^{-1}; $M_{n,GPC}$ = N/A g mol^{-1}; $Đ$ = N/A (GPC analysis gave unreliable values).

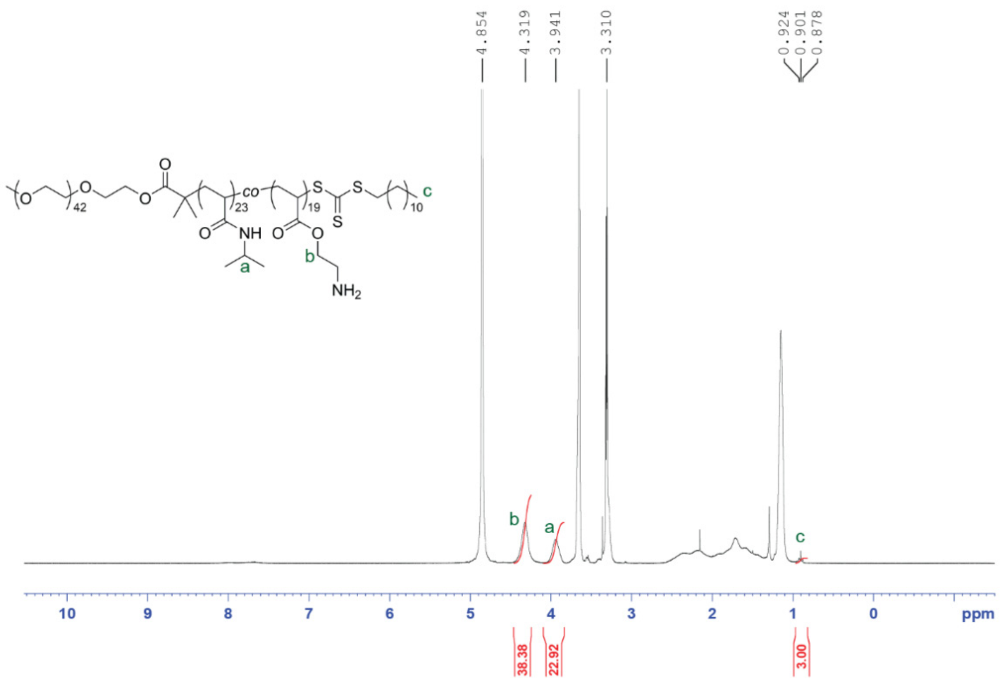

Supplementary Figure 45. ^{1}H NMR spectrum of PEG$_{43}$-*b*-P(NIPAM$_{23}$-*co*-AEA$_{19}$) in MeOD.

Synthesis of PEG$_{43}$-b-P(NIPAM$_{23}$-co-PDMI$_{19}$)

The *t*-Boc deprotected diblock terpolymer *PEG$_{43}$-b-P(NIPAM$_{23}$-co-AEA$_{19}$)* (80.5 mg, 0.011 mmol), *N*-(pentafluorophenyl butyl ester)-perylene-3,4,9,10-tetracarboxylic monoimide dibutyl ester (PDMI-PFP, 203 mg, 0.267 mmol) and *N*,*N*-diisopropylethylamine (35.9 mg, 0.278 mmol) were dissolved in 10 mL of *N*,*N*-dimethylformamide and stirred under nitrogen at 40 °C. Progress of the reaction was monitored by TLC (dichloromethane:acetone, 9:1, v/v). After 48 h, the crude reaction mixture was separated into two equal portions (to avoid overloading of column) and was purified by size-exclusion chromatography (Bio-Beads S-X1,

THF). The initial red elution band corresponding to a higher molecular weight product was collected and further purified by dialysis (3.5 kDa MWCO) against THF:ethanol (1:1, v/v) followed by dichloromethane:ethanol (1:1, v/v). Further purification by dialysis was necessary due to the high tendency of unreacted PDMI-PFP to aggregate with the desired product, causing it to co-elute in the initial red elution band. The dialyzed product was then concentrated *in vacuo* and further dried under high vacuum to yield *PEG$_{43}$-b-P(NIPAM$_{23}$-co-PDMI$_{19}$)* as a dark red solid (182 mg, 88%). $M_{n,NMR}$ = 18,259 g mol^{-1}; $M_{n,GPC}$ = 16,100 g mol^{-1}; $Đ$ = 1.09.

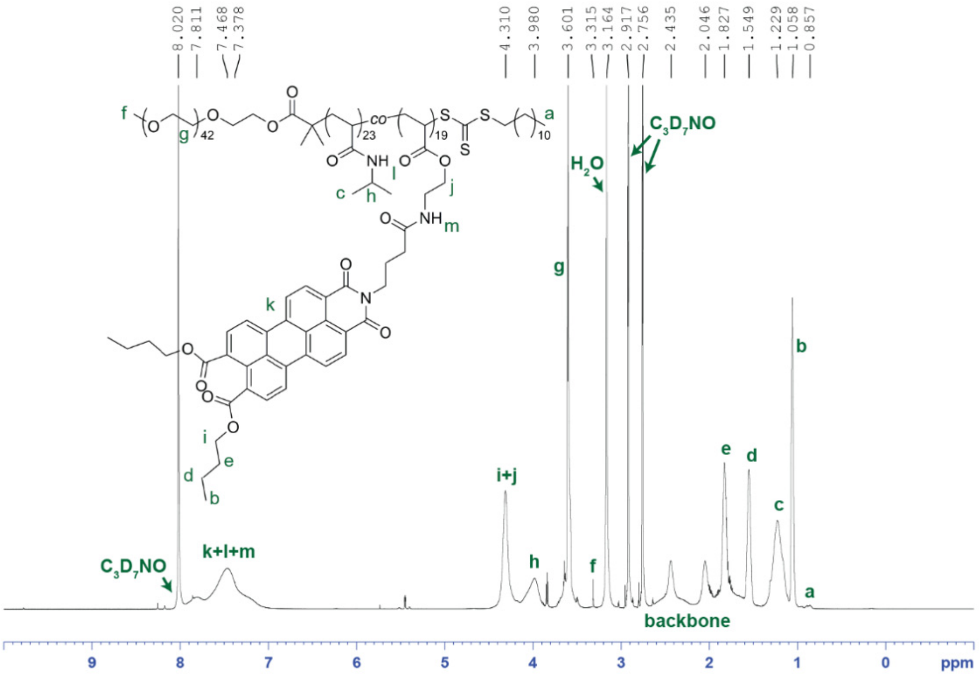

Supplementary Figure 46. ^{1}H NMR spectrum of PEG$_{43}$-*b*-P(NIPAM$_{23}$-*co*-PDMI$_{19}$) in C_3D_7NO measured at 70 °C.

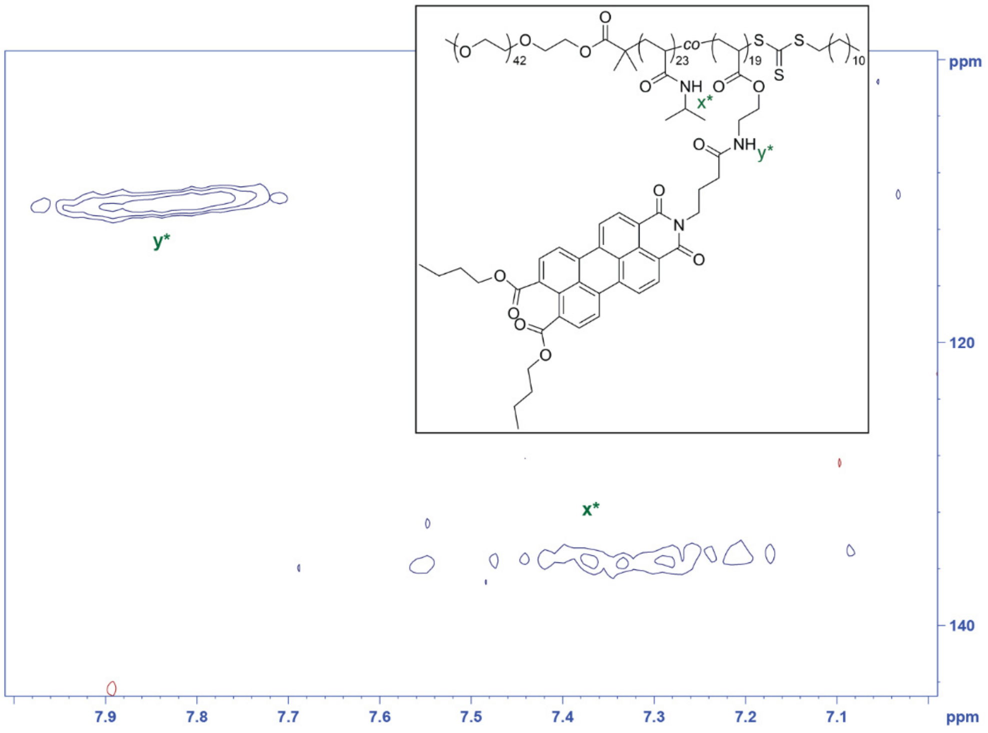

Supplementary Figure 47. ^1H-^{15}N HSQC NMR spectrum of PEG43-*b*-P(NIPAM23-*co*-PDMI19) in C_3D_7NO (measured at 70 °C) showing cross-peaks corresponding to the two different amide protons of the diblock terpolymer.

Supplementary Table 4. Integration values and relative integration values for ^1H-^{15}N cross-peaks **x*** and **y*** shown in Supplementary Fig. 47. The degree of dye functionalization on PEG43-*b*-P(NIPAM23-*co*-PDMI19) was obtained by multiplying the known number of repeating units of NIPAM (i.e. 23 repeat units, determined using integrals from the ^1H NMR spectrum (Supplementary Fig. 45) of PEG43-*b*-P(NIPAM23-*co*-AEA19)) by the relative integration value of 0.86 (*i.e.* 23 × 0.86 = 19.8 ≈ 19 repeat units of PDMI).

Peak	Integral (abs)	Integral (rel)	ν(F2) (ppm)	ν(F1) [ppm]
**y***	4.58E+06	0.86	7.84	110.1
**x***	5.33E+07	1.00	7.39	135.2

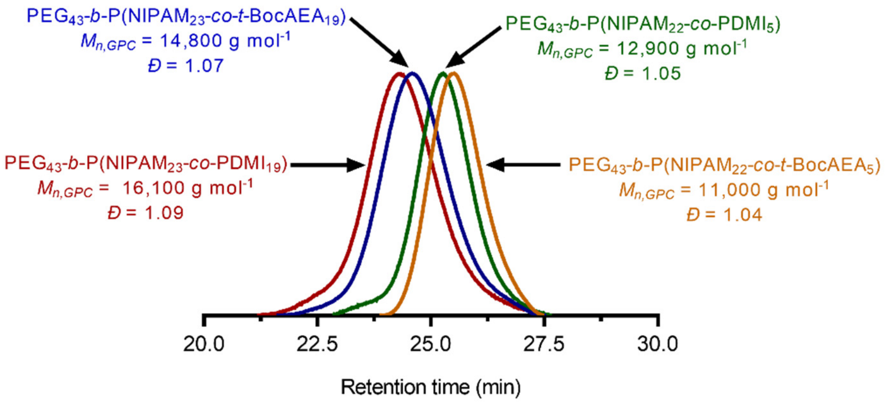

Supplementary Figure 48. DMAc GPC traces of PEG_{43}-*b*-P($NIPAM_{22}$-*co*-*t*-$BocAEA_5$), PEG_{43}-*b*-P($NIPAM_{23}$-*co*-*t*-$BocAEA_{19}$), PEG_{43}-*b*-P($NIPAM_{22}$-*co*-AEA_5), PEG_{43}-*b*-P($NIPAM_{23}$-*co*-AEA_{19}), PEG_{43}-*b*-P($NIPAM_{22}$-*co*-$PDMI_5$) and PEG_{43}-*b*-P($NIPAM_{23}$-*co*-$PDMI_{19}$).

Supplementary References

1. Webb, J. E. A. *et al.* Quantifying highly efficient incoherent energy transfer in perylene-based multichromophore arrays. *Phys. Chem. Chem. Phys.* **18**, 1712–1719 (2016).
2. Xue, C., Sun, R., Annab, R., Abadi, D. & Jin, S. Perylene monoanhydride diester: a versatile intermediate for the synthesis of unsymmetrically substituted perylene tetracarboxylic derivatives. *Tetrahedron Lett.* **50**, 853–856 (2009).
3. Ho, H. T., Pascual, S., Montembault, V., Casse, N. & Fontaine, L. Innovative well-defined primary amine-based polyacrylates for plasmid DNA complexation. *Polym. Chem.* **5**, 5542–5545 (2014).
